# Noncanonical Folding of Peptoid Oligomers: Formation of a Closed Conformation in Nonpolar Solvent

**DOI:** 10.1021/acs.orglett.6c02040

**Published:** 2026-06-23

**Authors:** Jinyoung Oh, Min June Yang, Xingyu Chen, Juhye Shin, Bradley S. Harris, Robert M. Raddi, Suhyun Park, Marcel D. Baer, Hohjai Lee, Chin-Ju Park, Vincent A. Voelz, Jiwon Seo

**Affiliations:** † Department of Chemistry, 65419 Gwangju Institute of Science and Technology, 123 Cheomdangwagi-ro, Buk-gu, Gwangju 61005, Republic of Korea; ‡ Department of Chemistry, College of Science and Technology, 6558 Temple University, 1801 N. Broad Street, Philadelphia, Pennsylvania 19122, United States; § Physical and Computational Sciences Directorate, 6865 Pacific Northwest National Laboratory, Richland, Washington 99354, United States

## Abstract

Conformational behavior of peptoids in low-dielectric solvents remains poorly understood despite its relevance to membrane environments. Here, conformations of *N*-(*S*)-1-phenylethylglycine (*N*spe) homo-oligomers were investigated in chloroform using NMR spectroscopy and MD simulations. *N*spe_7_ populated two closed conformations, while *N*spe_10_ adopted a single conformation reminiscent of the *N*spe_9_ threaded-loop structure. End-to-end hydrogen bonding and hydrophobic side-chain shielding stabilize these compact folds, minimizing polar surface area. These findings provide insights into peptoid folding in nonpolar media and solvent-directed conformational switching.

## Graphical abstract

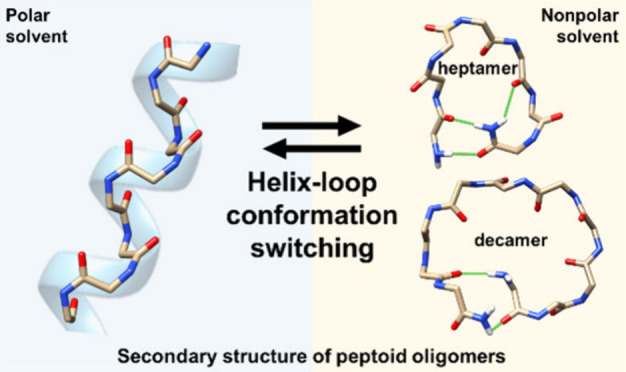

Elucidating the sequence–structure relationship in peptidomimetic foldamers is critical to expanding the reach of predictive AI models into non-natural chemical spaces. Peptoids are a representative class of peptidomimetics based on oligo-*N*-substituted glycines. The side chains of peptoids are attached on the backbone amide nitrogen rather than on the α-carbon. This tertiary amide backbone prevents conventional N–H···O intramolecular hydrogen bonds—a main interaction during secondary structure folding in natural peptides—and yields distinct folding behavior.^1–4^ The modular synthetic protocol enables diverse side-chain incorporation with tunable conformational and physicochemical properties.^5–8^

Because the peptoid backbone possesses intrinsic conformational flexibility mainly due to isoenergetic *cis*/*trans* isomerism,^9,10^ the design and prediction of secondary or tertiary structures remain a major challenge. Thus, strategies for constructing well-defined structural motifs represent a central focus of peptoid research.^6,7^ A widely adopted approach involves incorporating *Nα*-chiral monomers, most notably *N*-(*S*)-1-phenylethylglycine (*N*spe, Figure 1A), which promote the formation of polyproline type-I (PPI)-like helices with all *cis*-amide geometry.^1–4,11–13^ *N*-Terminal acetylation further stabilizes helical conformations by inducing *cis*-amide formation, resulting in increased folding propensity.^12–14^ Expanding beyond the well-established helix formation, diverse structural motifs have been identified. Notable examples include the “threaded-loop” conformation found in *N*spe nonamer in acetonitrile,^15^ and the peptoid “ribbon” structure found in alternating *cis*- and *trans*-forming monomers.^16^ Extended all-*cis* backbone geometries have been demonstrated across diverse crystalline peptoid nanostructures—including nanosheets,^17–20^ nanofibers,^19,21^ and peptoid polymer lattices^21,22^ —where all-*cis* arrangements translate into well-defined supramolecular assemblies. Such structural understandings of peptoids widen the scope of accessible peptoid architectures, facilitating the development of novel functional peptoids in drug discovery,^23–27^ bioinspired materials,^28–30^ and molecular recognition.^31–33^

**Figure 1:**
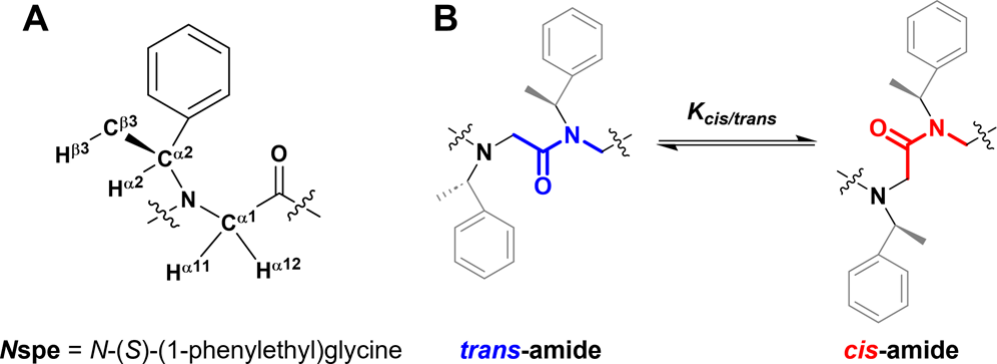
(A) Chemical structure and labels of *N*spe residues. (B) Backbone amide *cis*–*trans* isomerism.

Investigation of peptoid structures in nonpolar solvents is motivated by their direct relevance to biological membrane environments. Among the most impactful functions of peptoids is their interaction with lipid-bilayer membranes; the insertion of peptoids into the hydrophobic interior of these membranes leads to antimicrobial membrane disruption or cellular penetration.^34,35^ Despite this, very little is known about peptoid conformational preferences in low-dielectric media, leaving a critical gap in our mechanistic understanding. Chloroform (ε ≈ 4.8) serves as an established mimic of the lipid bilayer interior, making it an ideal model solvent for such studies. Among structural characterization methods, NMR techniques provide high-resolution mapping of *cis*-*trans* isomerization and detailed key spatial relationships between backbone and side chain protons.^3,4,15,16^ MD simulations further clarify the energetics and preferred structures in chloroform.

To examine the backbone folding behavior in chloroform, a series of *N*spe homo-oligomers ranging from trimer (*N*spe_3_) to dodecamer (*N*spe_12_) was systematically investigated. This series has been extensively characterized in polar solvents—particularly acetonitrile and methanol—where longer oligomers preferentially adopt PPI-like all-*cis* helical conformations.^1,2,4,11^ To evaluate the impact of a low-dielectric environment on folding, NMR spectroscopy and MD simulations were mainly employed. High-resolution NMR spectroscopy enabled sequence-specific assignment of *cis*-*trans* backbone amide conformations (Figure S3), and nuclear Overhauser effect (NOE) signals provided proton proximity restraints. These experimentally derived restraints guided MD simulations to construct and visualize conformational ensembles for each oligomer. Interestingly, the *N*spe heptamer (*N*spe_7_) and decamer (*N*spe_10_) exhibited unexpected structural features in chloroform: rather than adopting the anticipated PPI-like helices, these oligomers folded into distinct closed conformations. Overall, this study highlights how the hydrophobic environment drives *N*spe-based peptoid folding at requisite chain lengths, providing structural understanding in low-dielectric solvents.

Two-dimensional ^1^H–^1^H COSY, ^1^H–^13^C HSQC, and ^1^H–^1^H ROESY spectra enabled classification of methine proton resonances according to their association with *cis*-amide, *trans*-amide, or *N*-terminal residues. NMR studies of *N*spe homo-oligomers in chloroform revealed notably lower K_*cis*/*trans*_ values, with more than half of peptoids exhibiting K_*cis*/*trans*_ below 1 (Table 1), reflecting a preference for *trans*-amide bonds and overall increase in conformational heterogeneity.

**Table 1:** Overall *K*_*cis/trans*_ Values As Determined by Integration of the ^1^H–^1^H COSY Spectra in CDCl_3_ at 25 °C with a Sample Concentration of 50 mM

Peptoid	Sequence	*K*_*cis/trans*_ ^a^
*N* **spe**_**3**_	H–(*N*spe)_3_–NH_2_	2.49
*N* **spe**_**4**_	H–(*N*spe)_4_–NH_2_	1.31
*N* **spe**_**5**_	H–(*N*spe)_5_–NH_2_	0.56
*N* **spe**_**6**_	H–(*N*spe)_6_–NH_2_	0.58
*N* **spe**_**7**_	H–(*N*spe)_7_–NH_2_	1.47
*N* **spe**_**8**_	H–(*N*spe)_8_–NH_2_	0.49
*N* **spe**_**9**_	H–(*N*spe)_9_–NH_2_	1.11
*N* **spe**_**10**_	H–(*N*spe)_10_–NH_2_	0.77
*N* **spe**_**11**_	H–(*N*spe)_11_–NH_2_	0.62
*N* **spe**_**12**_	H–(*N*spe)_12_–NH_2_	0.85

a *K*_*cis*/*trans*_ values represent the average over all backbone amide positions within each oligomer, as determined by integration of total methine proton cross-peak volumes associated with *cis*- and *trans*-amide conformers in ^1^H–^1^H COSY spectra. All oligomers were studied as their *N*-terminal protonated TFA salts.

To further investigate amide conformational preferences, detailed structural analysis was performed, focusing on the oligomers exhibiting well-resolved spectral features. Peptoid homo-oligomers *N*spe_3_, *N*spe_7_, *N*spe_9_, and *N*spe_10_ displayed sufficiently dispersed cross-peaks in ^1^H–^1^H COSY, ^1^H–^13^C HSQC, ^1^H–^13^C HMBC, and ^1^H–^1^H ROESY spectra (Figures S4–S7), enabling sequential assignments (Figure S3) and backbone amide isomerism determination.

## *N*spe_3_ (*N*spe Trimer)

*N*spe_3_ contains two amide bonds; the isomeric states (*cis* or *trans*) are assigned sequentially from the *N*-terminus (i.e., *ct* refers to the first amide as *cis* and the second as *trans*). All four theoretically possible conformations—*cc*, *ct*, *tc*, *tt*—were observed through 2D NMR spectroscopy (Figure S8). The population of each conformer was determined by integration of ^1^H–^1^H COSY cross-peaks, indicating that the *cc* conformation was major (54%), followed by *ct* (19%), *tc* (15%), and *tt* (12%, Table S4).

## *N*spe_7_ (*N*spe Heptamer)

*N*spe homo-oligomers longer than a tetramer have been reported to adopt PPI-like helices with all *cis*-amide bonds in acetonitrile or methanol.^4^ However, NMR investigations of *N*spe_7_ in chloroform revealed a distinct conformational outcome (Figure 2). *N*spe_7_ adopts two discrete folded conformations in chloroform, evidenced by two complete and well-dispersed sets of cross-peaks (Figure 2B). Sequential assignments, ordered from the *N*-terminus, were guided by through-bond and through-space correlations (Figure 2A). Both conformers exhibit significant *trans*-amide content, indicating different structural motifs compared with canonical PPI-like helices.

**Figure 2:**
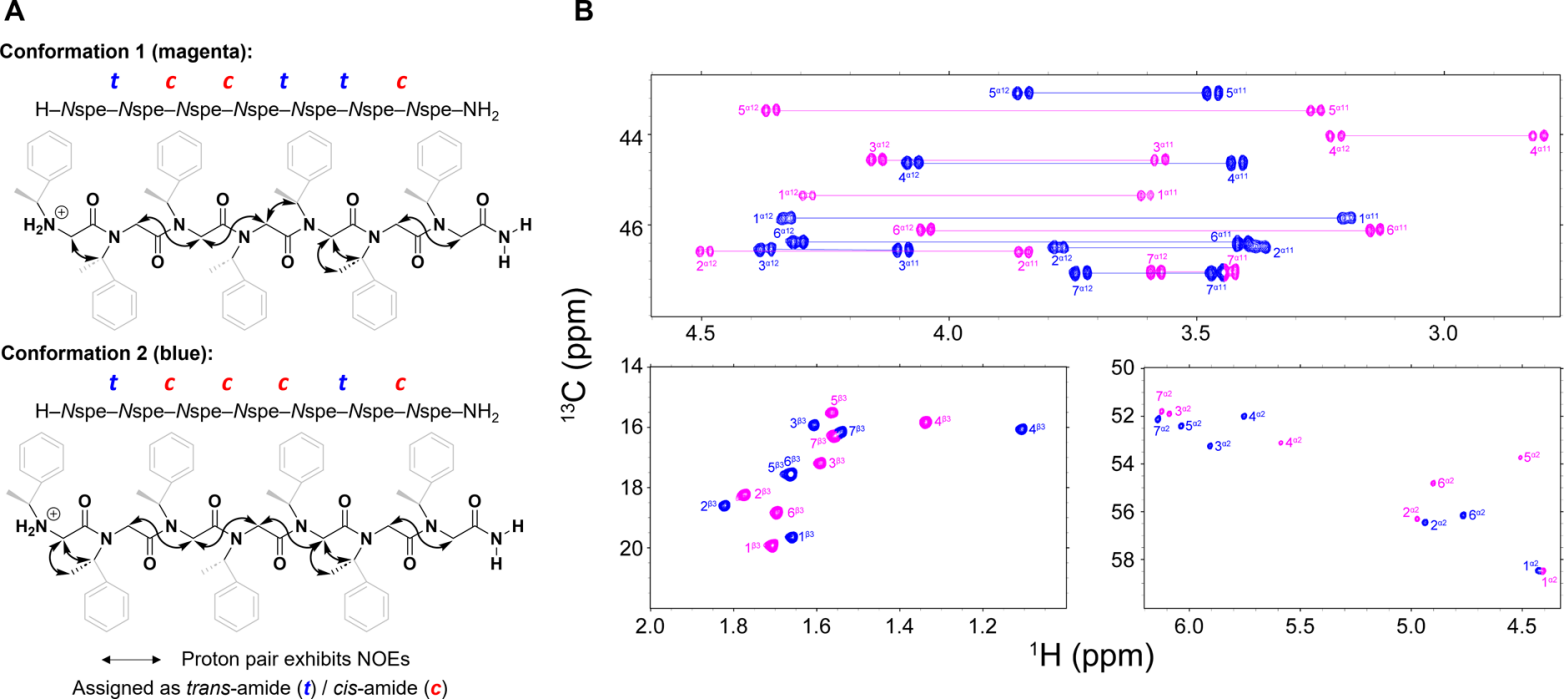
(A) Two distinct folded conformations of *N*spe_7_. (B) Expanded plots of ^1^H–^13^C HSQC spectra in CDCl_3_ at 25 °C with a 50 mM sample concentration.

The two conformations differ primarily in the amide isomerism between the fourth and fifth residues from the *N*-terminus, with relative populations of 55% and 38% for Conformations 1 and 2, respectively. A long-range NOE signal was observed between the sixth residue backbone alpha proton and the *C*-terminal amide proton, implying a noncovalent interaction that brings the rolled-up chain termini into proximity with an inner residue.

To construct 3D ensemble structures of the peptoid, NMR-integrated MD simulations were conducted (Figure 3). Hamiltonian replica exchange simulations were first performed for 200 ns to generate starting structures consistent with ω-angles biased under NMR-derived restraints (Tables S7–S10, Figure S14). Subsequently, these structures served as inputs for temperature replica-exchange MD (Figures S15–S20), followed by binning of samples from the lowest-temperature replica into conformation states uniquely defined by their backbone dihedral angles. Populations of these states were reweighted against NMR distance restraints using the BICePs algorithm, which infers the populations most consistent with the NMR data under explicit treatment of experimental uncertainty (Table S12, Figures S21–S27).^36,37^ The highest-population states in the resulting ensembles reveal specific intramolecular hydrogen bonds that stabilize the observed compact folds. Conformation 1 displayed hydrogen bonding between the *N*-terminal protonated amine and the *C*-terminal carbonyl (1.99 Å), the *N*-terminal carbonyl and the *C*-terminal amide proton (1.81 Å), and the fifth residue carbonyl and the *C*-terminal amide proton (2.69 Å, Figure 3E). Conformation 2 exhibited analogous interactions via similar hydrogen bonds, distances of 2.39 Å, 1.96 Å, and 2.49 Å, respectively (Figure 3F). These observations establish that end-to-end interactions collectively stabilize compact, nonhelical folds of *N*spe_7_ in the nonpolar environment. The specific *cis*/*trans*-amide arrangement (e.g., *tccttc* of Conformation 1) serves as the geometric scaffold that positions the chain termini in close proximity—the heptameric chain length proving particularly suited to provide the appropriate end-to-end distance and orientation for terminal hydrogen bond formation. The critical role of hydrogen bonding was further evidenced by dimethyl sulfoxide (DMSO) titration (Figure S11). Disruption of the hydrogen-bonding network by the addition of DMSO led to spectral patterns similar to those observed in acetonitrile, indicating a return to the helical conformation in polar environments.

**Figure 3:**
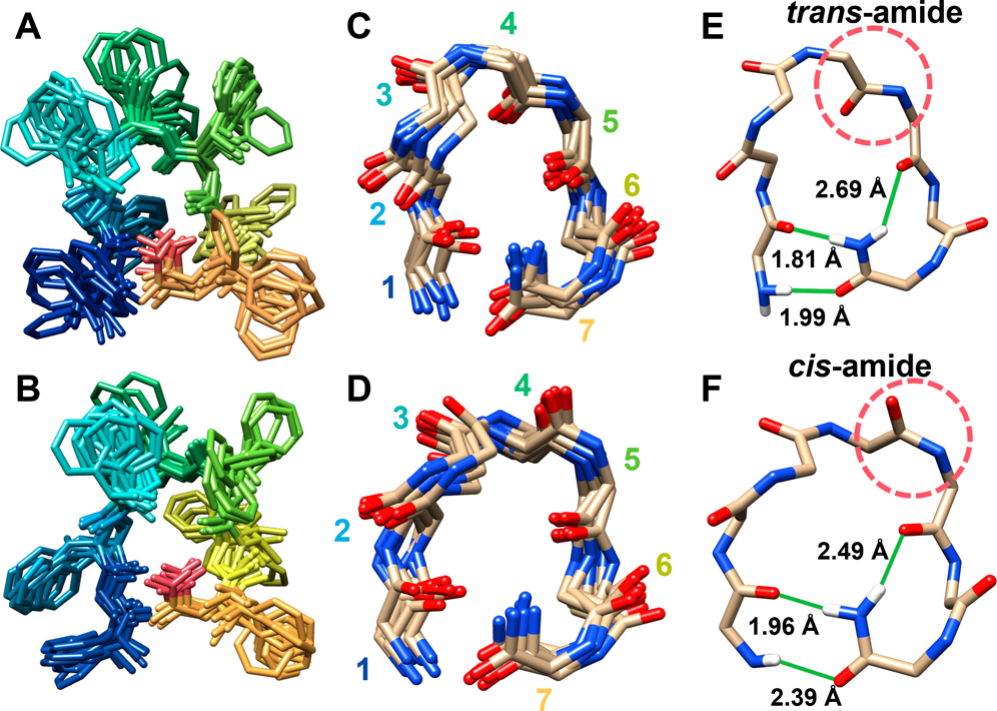
Solution structures of *N*spe_7_ in chloroform generated by NMR-integrated MD simulations. Ten structures from the BICePs-weighted most populated cluster are shown after best-fit superposition of Conformation 1 (A) and 2 (B), color-ramped from the *N*-terminus (blue) to *C*-terminus (red). Backbone cluster ensemble representations of Conformations 1 (C) and 2 (D). Representative structures from the ensembles of Conformation 1 (E) and 2 (F). Green lines indicate intramolecular hydrogen bonds.

## *N*spe_10_ (*N*spe Decamer)

Previously, Barron and co-workers discovered that *N*spe_9_ adopted a well-defined threaded-loop conformation in acetonitrile.^15^ This finding expanded the known repertoire of peptoid secondary structures by demonstrating that stable, nonhelical folds can arise from backbone isomerism and cooperative intramolecular interactions. Interestingly, *N*spe_10_ exhibited a homogeneous conformation in chloroform, and its structural feature was comparable to that of *N*spe_9_. NMR spectra of *N*spe_9_ and *N*spe_10_ revealed a single set of well-dispersed ^1^H–^1^H COSY and ^1^H–^13^C HSQC cross-peaks at 25 °C and 50 mM concentration (Figure 4B and Figures S4 and S5), indicating the presence of a single dominant conformer in solution (populations of 95% and 88% for *N*spe_9_ and *N*spe_10_, respectively).

**Figure 4:**
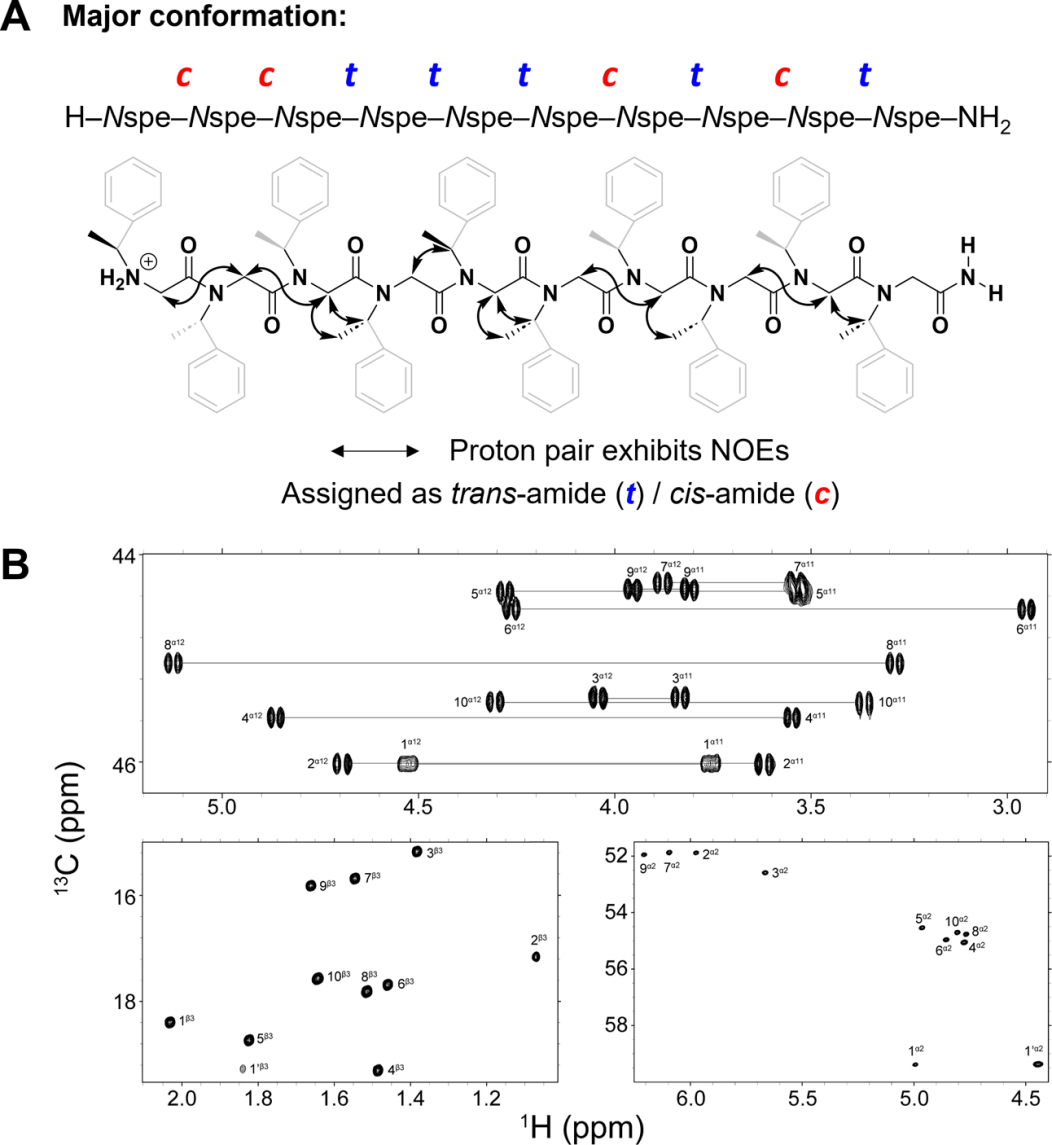
(A) Distinct folded conformation of *N*spe_10_. (B) Expanded plots of ^1^H–^13^C HSQC spectra in CDCl_3_ at 25 °C with a 50 mM sample concentration.

The detailed amide isomerism of *N*spe_10_ (Figure 4A) features a distinctive arrangement of *cis*- and *trans*-amide bonds that matches the previously reported *N*spe_9_ threaded-loop^15^ in identical order, differing only by an additional *C*-terminal *trans*-amide. Long-range NOE correlations between nonadjacent residues (Figure 5A)—especially between 10^α11^ and 1^α12^; 7^β3^ and 1^β3^—further support the presence of this closed-loop conformation. Complementary MD simulations identified a key intramolecular hydrogen bond (Figure 5D) between the *N*-terminal protonated amine proton and the ninth residue carbonyl (2.10 Å), and the *N*-terminal carbonyl and the *C*-terminal amide proton (2.34 Å). Bulky hydrophobic side chains, particularly from the third and ninth residues, masked the backbone polar functional groups, effectively reducing solvent-exposed polarity and stabilizing the folded structure. These results demonstrate that *N*spe_10_ adopts a compact end-to-end loop conformation in chloroform, stabilized cooperatively by intramolecular hydrogen bonding and hydrophobic side-chain shielding, with the *cis*/*trans*-amide arrangement serving as the geometric scaffold.

**Figure 5:**
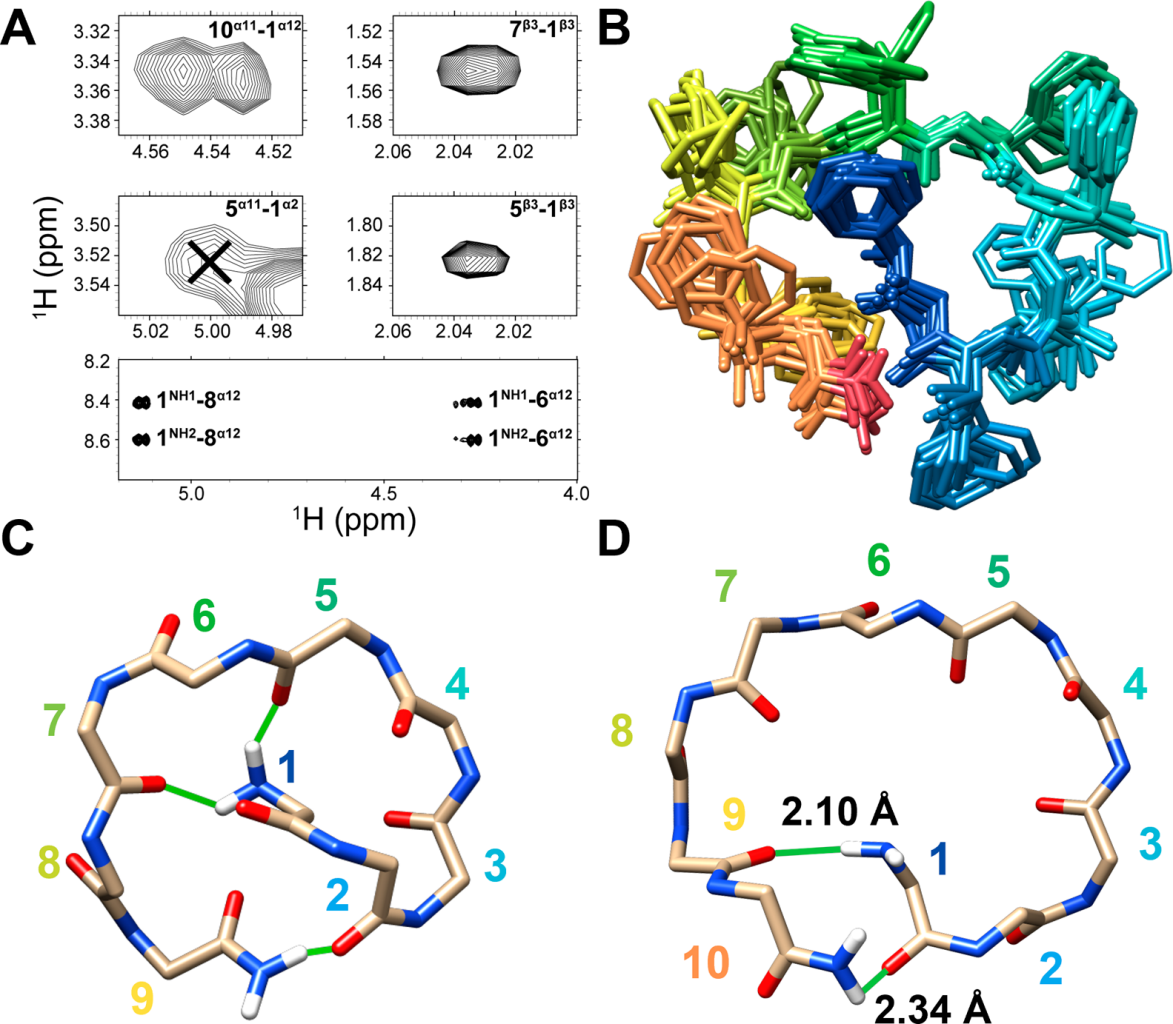
Solution structure of *N*spe_10_ in a chloroform environment. (A) ROESY cross-peaks indicating long-range NOEs (B) Ten structures following a best-fit superposition of the backbone atoms, color-ramped from *N*-terminus (blue) to *C*-terminus (red). (C) Previously reported *N*spe_9_ threaded-loop in acetonitrile,^15^ reconstructed from reported dihedral angles. (D) Representative *N*spe_10_ structure from the backbone cluster ensemble. Green lines indicate intramolecular hydrogen bonds.

In summary, this work reports the conformational preferences of *N*spe peptoid oligomers in chloroform. In contrast to all *cis*-amide PPI-like helices, *N*spe homo-oligomers in low-dielectric media exhibited decreased conformational homogeneity with notably lower *K*_*cis*/*trans*_ values. However, *N*spe_7_ and *N*spe_10_ formed well-defined conformers stabilized by end-to-end intramolecular hydrogen bonding—with the protonated *N*-terminus serving as a key hydrogen-bond donor^15,38^ —and hydrophobic side-chain shielding. This terminal hydrogen bonding, common to all folded conformations here, may offer a general strategy for stabilizing linear peptoid structures in nonpolar environments.

These closed conformations reduce solvent-exposed polarity in nonpolar media, whereas helical and open conformations maximize solubility in polar media. Consistent with this polar surface masking, *N*spe_7_ exhibited marginal passive membrane permeability in parallel artificial membrane permeability assays (PAMPA; Table S6). This chameleonic behavior is characteristics of macrocycles with passive membrane permeability, such as cyclosporin A.^39,40^ By demonstrating conformational adaptation of peptoids to environmental polarity—as evidenced by the DMSO titration (Figures S11–S13)—our findings offer deeper structural insights into peptoid foldamers. Given that polar surface masking and polarity-induced conformational switching are essential features of chameleonic macrocycles, translating these traits into peptoid design could yield versatile, solvent-responsive peptoid systems. Such advancements hold promise for the development of environment-sensitive conformational-switching sensors,^41^ molecular recognition systems,^42^ membrane-active peptoids that partition into the bilayer core,^34,35^ and passively membrane-permeable peptoids capable of modulating intracellular protein–protein interactions.

## Supplementary Material



## Data Availability

The data underlying this study are available in the published article and its Supporting Information.

## Notes

Abbreviations, synthesis of peptoids, experimental procedures, simulation details, analytical HPLC data, HRMS data, and NMR spectra of compounds (PDF)
