# Supplementary material for: Noncanonical Folding of Peptoid Oligomers: Formation of a Closed Conformation in Nonpolar Solvent
Source: Org Lett. 2026 Jun 23;28(26):8375–81. doi: 10.1021/acs.orglett.6c02040 (PMC13339750; doi:10.1021/acs.orglett.6c02040)
Supplement: Supplementary file 1 [file ol6c02040_si_001.pdf]

Supporting Information

Non-canonical folding of peptoid oligomers: Formation of a closed conformation in nonpolar solvent

Jinyoung Oh,[a,†] Min June Yang,[a,†] Xingyu Chen,[b,†] Juhye Shin,[a] Bradley S. Harris,[c] Robert M. Raddi,[b] Suhyun Park,[a] Marcel D. Baer,[c] Hohjai Lee,[a,*] Chin-Ju Park,[a,*] Vincent A. Voelz,[b,*] Jiwon Seo.[a,*]

[a]Department of Chemistry, Gwangju Institute of Science and Technology, 123 Cheomdangwagi-ro, Buk-gu, Gwangju, 61005, Republic of Korea.
[b]Department of Chemistry, College of Science and Technology, Temple University, 1801 N. Broad Street, Philadelphia, Pennsylvania 19122, United States.
[c]Physical and Computational Sciences Directorate, Pacific Northwest National Laboratory, Richland, WA 99354, United States.
*jseo@gist.ac.kr (J. Seo), voelz@temple.edu (V. A. Voelz), cjpark@gist.ac.kr (C.-J. Park), hohjai@gist.ac.kr (H. Lee)

†These authors contributed equally to this work.

Table of Contents

1. Abbreviations S5

2. Methods S5

(1) General method S5

(2) Peptoid synthesis S6

(3) NMR analysis S6

(4) DFT calculation S7

(5) Parallel artificial membrane permeability assay (PAMPA) S7

3. Characterization S9

Figure S1. HPLC chromatograms and MS spectra of *N*spe$_3$–*N*spe$_7$. The HPLC chromatograms were monitored at 220 nm and MS spectra were measured by LC/Q-Orbitrap MS S9

Figure S2. HPLC chromatograms and MS spectra of *N*spe$_8$–*N*spe$_{12}$. The HPLC chromatograms were monitored at 220 nm and MS spectra were measured by LC/Q-Orbitrap MS S10

4. NMR analyses S11

(1) Sequential assignment S11

Figure S3. Sequential assignment of peptoid residues using 2D NMR spectroscopy S11

(2) ^{1}H–^{13}C HSQC spectra S12

Figure S4. C$^{\alpha2}$-H$^{\alpha2}$ peaks from peptoids in ^{1}H–^{13}C HSQC spectra S12

(3) 2D NMR spectra of *N*spe$_4$, *N*spe$_5$, *N*spe$_6$, *N*spe$_8$, *N*spe$_{11}$ and *N*spe$_{12}$ S13

Figure S5. Expanded ^{1}H–^{1}H COSY spectra of *N*spe$_4$, *N*spe$_5$, *N*spe$_6$, *N*spe$_8$, *N*spe$_{11}$ and *N*spe$_{12}$ in CDCl$_3$ at 25 °C, recorded at a sample concentration of 50 mM S13

Figure S6. Comparison of COSY and ROESY Spectra for *N*spe$_9$ and *N*spe$_{10}$, *N*spe$_{11}$ and *N*spe$_{12}$ peptoids S14

(4) Terminal protons S15

Figure S7. Assignment of the *C*-terminal amide (–CONH$_2$) resonances of the *N*spe$_7$ (left) and the *N*-terminal amine (–NH$_2^+$) resonances of the *N*spe$_{10}$ (right) by 2D NMR spectroscopy S15

(5) Chemical shift of *N*spe$_3$, *N*spe$_7$, *N*spe$_9$ and *N*spe$_{10}$ S16

Table S1. Chemical shifts of *N*spe$_3$ at 900 MHz in CDCl$_3$ S16

Table S2. Chemical shifts of *N*spe$_7$ at 900 MHz in CDCl$_3$ S16

Table S3. Chemical shifts of *N*spe$_9$ and *N*spe$_{10}$ at 900 MHz in CDCl$_3$ S17

(6) Conformational analysis of *N*spe$_3$ S18

Figure S8. NMR-based conformational analysis of *N*spe$_3$. Expanded plots of (A) ^{1}H–^{13}C HSQC spectra and (B) ^{1}H–^{1}H ROESY in CDCl$_3$ at 25 °C with a sample concentration of 50 mM S18

Table S4. *N*spe$_3$ population table S18

Figure S9. Experimental ^{1}H NMR chemical shifts of the H$^{\alpha11}$, H$^{\alpha12}$, and H$^{\alpha2}$ for the four *N*spe$_3$ isomers .. S19

Figure S10. Correlation between calculated and experimental chemical shifts for all targeted protons, with the dotted line showing the linear fit. Correlation plot focusing on the H$^{\alpha2}$ S19

(7) DMSO titration S19

Figure S11. Effect of DMSO-*d*$_6$ addition on the ^{1}H–^{1}H COSY (top) and ^{1}H–^{13}C HSQC (bottom) spectra of the *N*spe$_7$ at 25 °C. Overlay of COSY spectra recorded at 600 MHz in CDCl$_3$ containing 0%, 9%, 33% DMSO-*d*$_6$, and CD$_3$CN S20

Figure S12. Effect of DMSO-d_6 addition on the ^{1}H–^{1}H COSY (top) and ^{1}H–^{13}C HSQC (bottom) spectra of the *N*spe$_{10}$ at 25 °C. Overlay of COSY spectra recorded at 600 MHz in $CDCl_3$ containing 0%, 9%, and 33% DMSO-d_6 .... S20

Figure S13. Effect of D_2O addition on the ^{1}H–^{1}H COSY (top) and ^{1}H–^{13}C HSQC (bottom) spectra of the *N*spe$_{10}$ at 25 °C. Overlay of COSY spectra recorded at 600 MHz in CD_3CN containing 0%, 9%, 33% and 50% D_2O S21

(8) Variable temperature (VT) NMR S22

Figure S14. Temperature-dependent ^{1}H–^{1}H COSY spectra of the *N*spe$_7$ and the *N*spe$_{10}$ at 5 °C (blue) and 45 °C (red). Overlay of COSY spectra recorded at 600 MHz in $CDCl_3$ S22

(9) Competing interactions drive solvent-dependent folding S22

5. Polar surface area and permeability implications S23

Table S5. Topological and conformational PSA calculation of *N*spe$_7$ and *N*spe$_{10}$ S23

Table S6. PAMPA permeability of *N*spe$_7$, *N*spe$_9$, and *N*spe$_{10}$ S23

6. MD simulation S24

(1) Overview S24

(2) Determination of NOE distance restraints S24

Table S7. Compute distance categories of interproton pairs for *N*spe$_{7\text{-}1}$ and *N*spe$_{7\text{-}2}$ S25

Table S8. Compute distance categories of interproton pairs for *N*spe$_{10}$ S26

(3) Molecular Simulation S27

(4) Hamiltonian Replica Exchange (HREX) simulations S27

Figure S15. Visualization of distance and omega dihedral angle restraint potentials S29

Table S9. Parameters used to define flat-bottom distance restraints for strong, medium, and weak distance restraints S29

Table S10. Summary of free-energy perturbation λ value for each peptoid used in HREX and REMD simulations S30

(5) Temperature Replica Exchange MD (REMD) simulations S31

Figure S16. Traces of thermodynamic index *i* over time for REMD simulations of *N*spe$_{7\text{-}1}$ S32

Figure S17. Traces of thermodynamic index *i* over time for REMD simulations of *N*spe$_{7\text{-}2}$ S32

Figure S18. Traces of thermodynamic index *i* over time for REMD simulations of *N*spe$_{10}$ S33

Figure S19. Traces of backbone omega-, phi- and psi-angles, and sidechain chi-angles, observed over time in the REMD simulation for each residue of *N*spe$_{7\text{-}1}$ in the unbiased ensemble at 300 K S34

Figure S20. Traces of backbone omega-, phi- and psi-angles, and sidechain chi-angles, observed over time in the REMD simulation for each residue of *N*spe$_{7\text{-}2}$ in the unbiased ensemble at 300 K S35

Figure S21. Traces of backbone omega-, phi- and psi-angles, and sidechain chi-angles, observed over time in the REMD simulation for each residue of *N*spe$_{10}$ in the unbiased ensemble at 300 K S36

(6) Conformational clustering and BICePs reweighting of simulated ensembles against experimental distance restraints S37

Table S11. BICePs models and parameters used for each system S38

Table S11. BICePs models and parameters used for each system S39

Figure S22. Mean distances and variances across conformational states binned by unique sets of omega- and phi-angles, compared to the experimental restraint distances, for *N*spe$_{7\text{-}1}$, *N*spe$_{7\text{-}2}$, and *N*spe$_{10}$ S40

Figure S23. Summaries of the BICePs results for *N*spe$_{7\text{-}1}$ S41

Figure S24. Summaries of the BICePs results for *N*spe$_{7\text{-}2}$ S41

Figure S25. Summaries of the BICePs results for *N*spe$_{10}$ S42

Figure S26. Structural characterization of the most populated conformational state for *N*spe$_{7\text{-}1}$ predicted by BICePs S43

Figure S27. Structural characterization of the most populated conformational state for *N*spe$_{7\text{-}2}$ predicted by BICePs S44

Figure S28. Structural characterization of the most populated conformational state for *N*spe$_{10}$ predicted by BICePs S45

7. References S46

1. Abbreviations

Acetonitrile (ACN); Becke three parameter Lee-Yang-Parr (B3LYP); Bayesian inference of conformational populations (BICePs); homonuclear correlation spectroscopy (COSY); density functional theory (DFT); *N,N*-diisopropyl carbodiimide (DIC); *N,N'*-dimethylformamide (DMF); dimethyl sulfoxide (DMSO); electrospray ionization mass spectrometry (ESI-MS); 9-fluorenylmethoxyl carbonyl (Fmoc); general AMBER force field 2 (GAFF2); gauge-including atomic orbital (GIAO); heteronuclear multiple bond correlation (HMBC); high-performance liquid chromatography (HPLC); Hamiltonian replica exchange (HREX); heteronuclear single quantum coherence spectroscopy (HSQC); linear constraint solver (LINCS); molecular dynamics (MD); nuclear Overhauser effect (NOE); isobaric-isothermal ensemble (NPT); canonical ensemble (NVT); *N*-methyl-2-pyrrolidone (NMP); parallel artificial membrane permeability assay (PAMPA); phosphate-buffered saline (PBS); particle mesh Ewald (PME); polar surface area (PSA); rotating-frame Overhauser enhancement spectroscopy (ROESY); solvent-accessible surface area (SASA); solvent model based on density (SMD); systematic and extensible force field for peptoids (STEPs); trifluoroacetic acid (TFA); triisopropylsilane (TIS); tetramethylsilane (TMS); topological polar surface area (TPSA); variable-temperature NMR (VT NMR).

2. Methods

(1) General methods

All reagents were purchased from Sigma-Aldrich (St. Louis, MO, USA), Beadtech (Ansan, Korea), Novabiochem (Merck KGaA, Darmstadt, Germany), TCI (Tokyo, Japan) or Cambridge Isotope Laboratories (Tewksbury, MA, USA), and they were used without further purification. Microwave-assisted synthesis of peptoids was performed on a CEM MARS multimodal microwave reactor equipped with a magnetic stirrer and a fiber-optic temperature probe (CEM Corp., Matthews, NC, USA). Peptoid oligomers were analyzed by a Waters HPLC system. The Waters HPLC was equipped with a reverse-phase column (SunFire C18, 4.6 × 250 mm, 5 μm), Waters 2489 UV/Visible detector, Waters 1525 Binary HPLC Pump, Waters 2707 Autosampler and Waters 5CH column oven. The mobile phases were water (A, +0.1% TFA) and ACN (B, +0.1% TFA). At 40 ℃, before sample was injected, the column was conditioned with 5% B for 10 min. After sample was injected, 5% B was kept for 2 min. The linear gradient of 5-100% B was applied over 30 min at a flow rate of 1 mL/min. UV/visible detector was set to monitor sample elution at 220 nm. Peptoid oligomers were purified by a preparative HPLC system (Waters PrepLC system, Waters 2489 UV/Visible detector, Waters fraction collector III) using C18 columns (SunFire C18, 19 × 150 mm, 5 μm and Phenomenex C18, 21.2 × 250 nm, 5 μm) at a flow rate of 14 mL/min. Sample elution was monitored by detection absorbance at 220 nm. The purity of the product fractions was confirmed by analytical HPLC, and fractions containing

pure product (>98% purity) were collected, lyophilized and stored at –80 °C. Mass data of peptoids were obtained from an Agilent LC/MS system which was equipped with 1260 Infinity LC and 6120 SQ bundle system with API-electrospray ion source.

(2) Peptoid synthesis

Using submonomer synthesis approaches with microwave heating, peptoid oligomers were synthesized under atmospheric pressure.[1] Rinkamide MBHA resin (0.65 mmol/g) was used. Typically, 0.16 mmol resin was swollen in CH_2Cl_2/DMF mixture for 30 min. The Fmoc deprotection was performed by adding a 20% solution of piperidine in DMF (v/v) at room temperature for 10 min. Then bromoacetylation was performed by adding bromoacetic acid (0.44 g, 3.2 mmol, 1.2 M in DMF) and DIC (0.41 g, 3.2 mmol, 510 μL) to the resin and irradiated at 35 ℃ (microwave, 400 W 50% power, ramp 30 sec, hold 1 min). For amine displacement, a 1.0 M solution of amine (3.2 mmol, in DMF) was added, and the reaction mixture was irradiated at 70 ℃ (microwave, 400 W 75% power, ramp 2 min, hold 1.5 min). After each step, DMF (3×), MeOH (2×) and CH_2Cl_2 (3×) were used to wash resin. Bromoacetylation and amine displacement were repeated until the desired peptoid sequence was obtained. Cleavage from the resin was performed with TFA/TIS/CH_2Cl_2 (95:2.5:2.5, v/v/v) at room temperature for 30 min. The cleavage solution was concentrated by a stream of nitrogen, diluted with ACN/H_2O (1:1, v/v) solution, and filtered by 0.45 μm Whatman Puradisc PTFE syringe filter (GE Healthcare's Life Sciences, Little Chalfont, Buckinghamshire, UK). The crude peptoid solution was purified, lyophilized, and analyzed by ESI-MS and analytical HPLC.

(3) NMR analysis

Peptoid oligomers Nspe$_3$–Nspe$_{12}$ were dissolved in $CDCl_3$ (99.8% D, stabilized) at a final concentration of 50 mM unless noted otherwise. For figures and $K_{cis/trans}$ analysis, samples were typically 50 mM in $CDCl_3$ at 25 °C. Solutions were loaded into 5 mm standard NMR tubes with a total volume of 500 μL. For DMSO titration, DMSO-d$_6$ (99.9% D, stabilized) was added directly to the 500 μL sample in the tube, using 50 μL or 250 μL additions, giving final volumes of 550 μL and 750 μL and approximately 9% and 33% DMSO-d$_6$ (v/v), respectively. All ^{13}C data were recorded at natural abundance.

NMR experiments were conducted on a Bruker AVANCE NEO 600 MHz spectrometer (GIST, Gwangju) equipped with a Prodigy probe and on a Bruker AVANCE III 900 MHz spectrometer (KBSI, Ochang) equipped with a cryogenic probe (Bruker, Billerica, MA, USA). Temperature was maintained at 25 °C unless otherwise specified, and variable-temperature experiments are noted where applicable. For spin-system assignments, two-dimensional ^{1}H–^{1}H homonuclear correlation spectroscopy (COSY, cosygpqf[2,3]), ^{1}H–^{13}C heteronuclear single quantum coherence (HSQC, hsqcedetgpsisp2.3[4,5]), and ^{1}H–^{13}C heteronuclear multiple bond correlation (HMBC, hmbcgplpndqf[6,7]) spectra were acquired. For structural analysis, ^{1}H–^{1}H rotating-frame Overhauser enhancement spectroscopy (ROESY,

roesyphpp.2[8,9]) spectra were recorded with a mixing time (τ_m) of 300 ms and a pulsed spin-lock (B_1) of 4.2 kHz. The ^{1}H carrier (o1p) was set to 4.0-4.5 ppm with a ^{1}H spectral width (SW) of 8-12 ppm. For the ^{13}C dimension, the SW and 13C carrier (o2p) were adjusted by region, and for targeted inspections very narrow ^{13}C windows were used to maximize digital resolution over specific areas. The recycle delay (d1) was 1-2 s, and the number of scans per increment (NS) was 4-16 to achieve adequate signal-to-noise ratio. Dummy scans (DS) followed sequence defaults unless specified. NMR data were processed in TopSpin (Bruker, Billerica, MA, USA) and analyzed in NMRFAM-POKY software.[10]

(4) Density functional calculations for NMR chemical shifts of Nspe$_3$

For each of the four possible *cis*/*trans*-amide isomers of the *N*spe$_3$, a conformational search starting from multiple initial geometries yielded at least five optimized structures. All geometry optimizations were performed at the B3LYP/6-31g+(d,p) level of theory, employing the SMD implicit solvent model for chloroform.[11] Subsequently, NMR chemical shifts for the optimized structures were calculated using the gauge-including atomic orbital (GIAO) method at the B97-2/6-31++g(2d,p) level.[12] Computed isotropic shielding constants were converted to chemical shifts by referencing them to the shielding constant of tetramethylsilane (TMS). The shielding constant for TMS was calculated at the identical level of theory, and no empirical scaling factors were applied. All calculations were carried out using the Gaussian 16 software package.[13]

To reduce computational cost, frequency calculations were performed only on conformers within an electronic energy window of 2.5 kcal/mol relative to the lowest-energy structure of each respective *cis*/*trans* isomer. The final predicted chemical shifts were determined by a Boltzmann-weighted average over this conformational ensemble except the structures with imaginary frequencies, based on their calculated thermal energies.[11]

(5) Parallel artificial membrane permeability assay (PAMPA)[14,15]

PAMPA was conducted with a 96-well donor plate with 0.45 μm hydrophobic immobilon-P membrane supports and a 96-well polystyrene acceptor plate in triplicate. A 1% (w/v) solution of soy lecithin in *n*-dodecane was prepared and sonicated for 5 min before being used for PAMPA. Lecithin solution (5 μL) was carefully added into each membrane support in the bottom of the donor plate without the pipet tip touching the membrane. Each sample was prepared as a 10 μM solution in 5% (v/v) DMSO/phosphate-buffered saline (PBS, pH = 7.4) buffer. The donor plates were prepared by the addition of a sample solution (150 μL), and the acceptor plates were filled with 5% DMSO/PBS buffer (300 μL). The donor plate was placed on top of the acceptor plate without any bubbles between the donor and acceptor plates. A lid was placed on the donor plate, and the whole plates were covered with a wet paper towel to prevent evaporation. Then, the plates were incubated for 18 h at room temperature. Next, an aliquot (50 μL) from each donor or acceptor was mixed with 2 μM Fmoc-Tyr(OtBu)-OH

dissolved in 1:1 (v/v) water/acetonitrile (50 µL) as an internal standard. The mixture (30 µL) was injected into the LC-MS instrument, and the analyte and internal standard were monitored in selected ion monitoring (SIM) mode. The analyte-to-standard peak area ratios were calculated and used to determine the relative concentrations. These concentrations were used to calculate the mass retention (R) and permeability (P_e)

$$R = 1 - \left(\frac{C_d \times V_d + C_a \times V_a}{C_0 \times V_d}\right) (1)$$

$$P_e = \frac{V_a \times V_d}{V_0 \times A \times t} \times ln\left(1 - \frac{C_a(V_d + V_a)}{C_d \times V_d + C_a \times V_a}\right) (2)$$

here C_0 is the initial concentration of donor plate (10 µM), C_a is the concentration of acceptor plate (µM), C_d is the concentration of donor plate (µM), V_a is the volume of acceptor plate (300 µL), V_d is the volume of donor plate (150 µL), A is the area of membrane (0.24 cm^2), t is the time (s), and $V_0 = V_a + V_d$.

3. Characterization

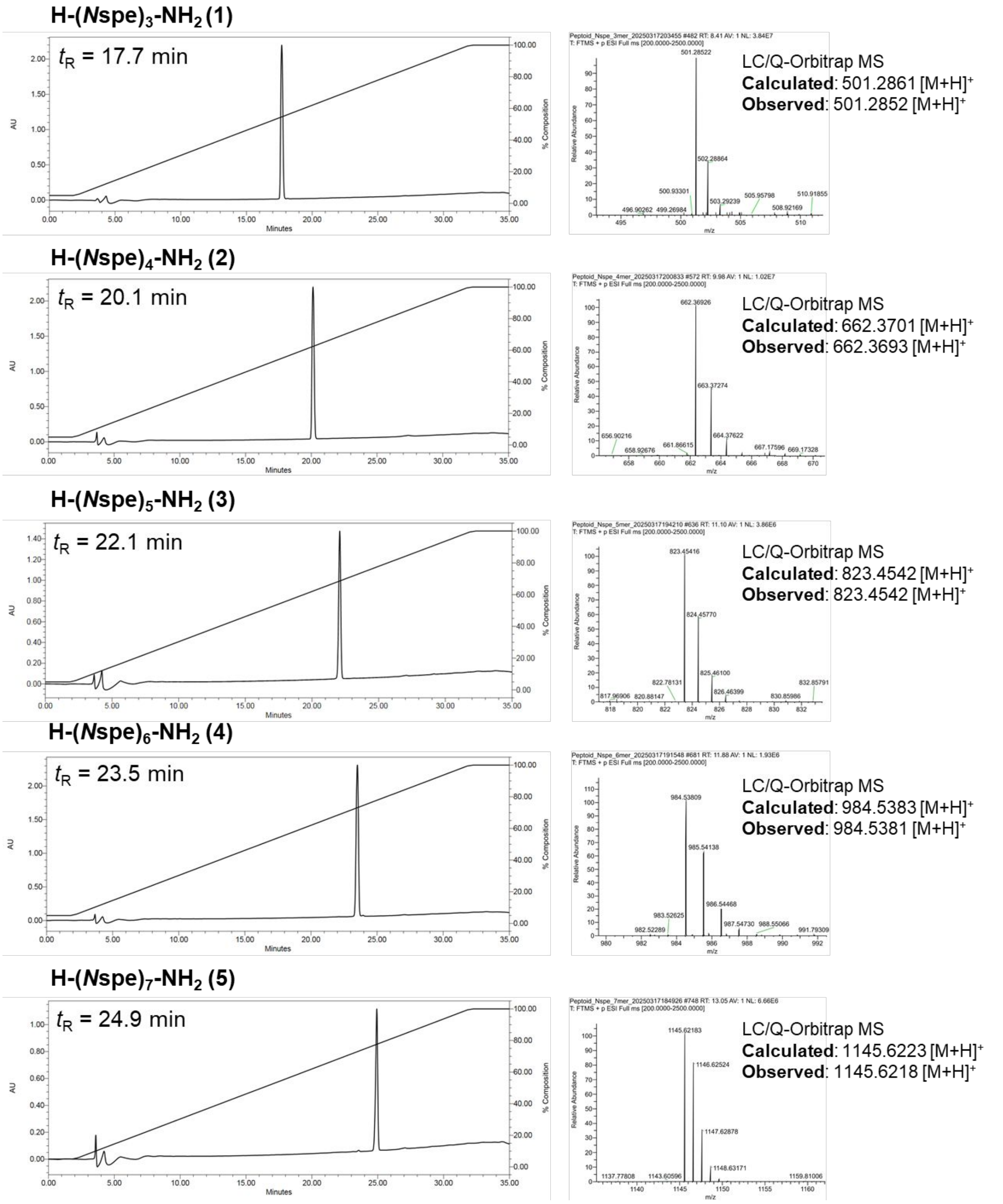

Figure S1. HPLC chromatograms and MS spectra of peptoids **1**–**5**. The HPLC chromatograms were monitored at 220 nm and MS spectra were measured by LC/Q-Orbitrap MS.

H-(*N*spe)$_8$-NH$_2$ (6)

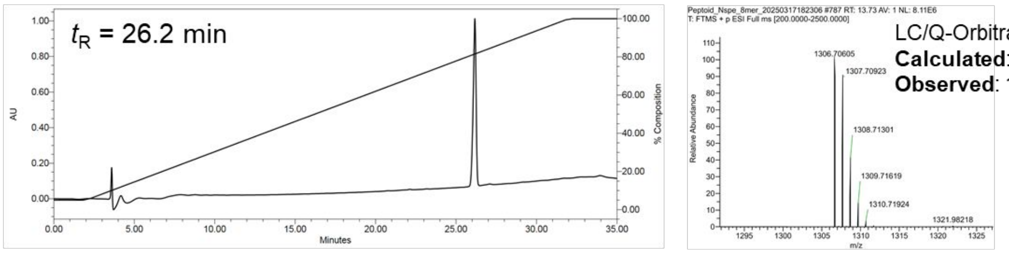

H-(*N*spe)$_9$-NH$_2$ (7)

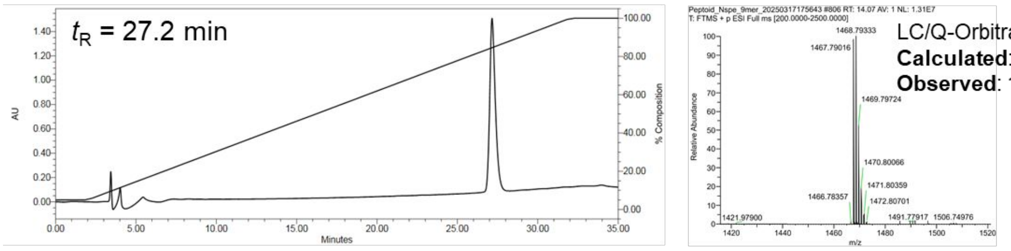

H-(*N*spe)$_{10}$-NH$_2$ (8)

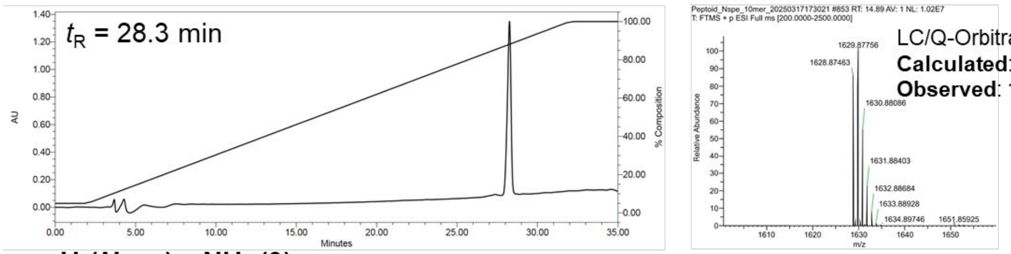

H-(*N*spe)$_{11}$-NH$_2$ (9)

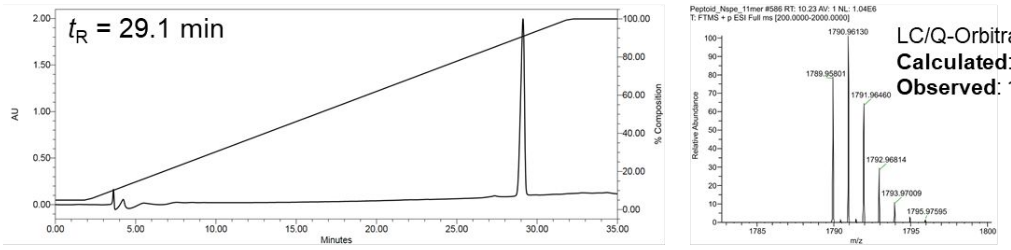

H-(*N*spe)$_{12}$-NH$_2$ (10)

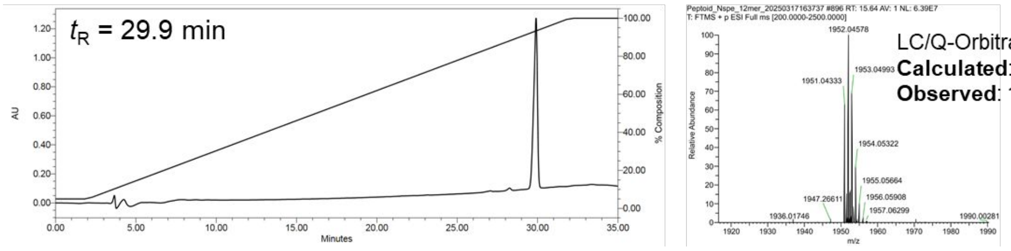

Figure S2. HPLC chromatograms and MS spectra of peptoids **6–10**. The HPLC chromatograms were monitored at 220 nm and MS spectra were measured by LC/Q-Orbitrap MS.

4. NMR analyses

(1) Sequential assignment

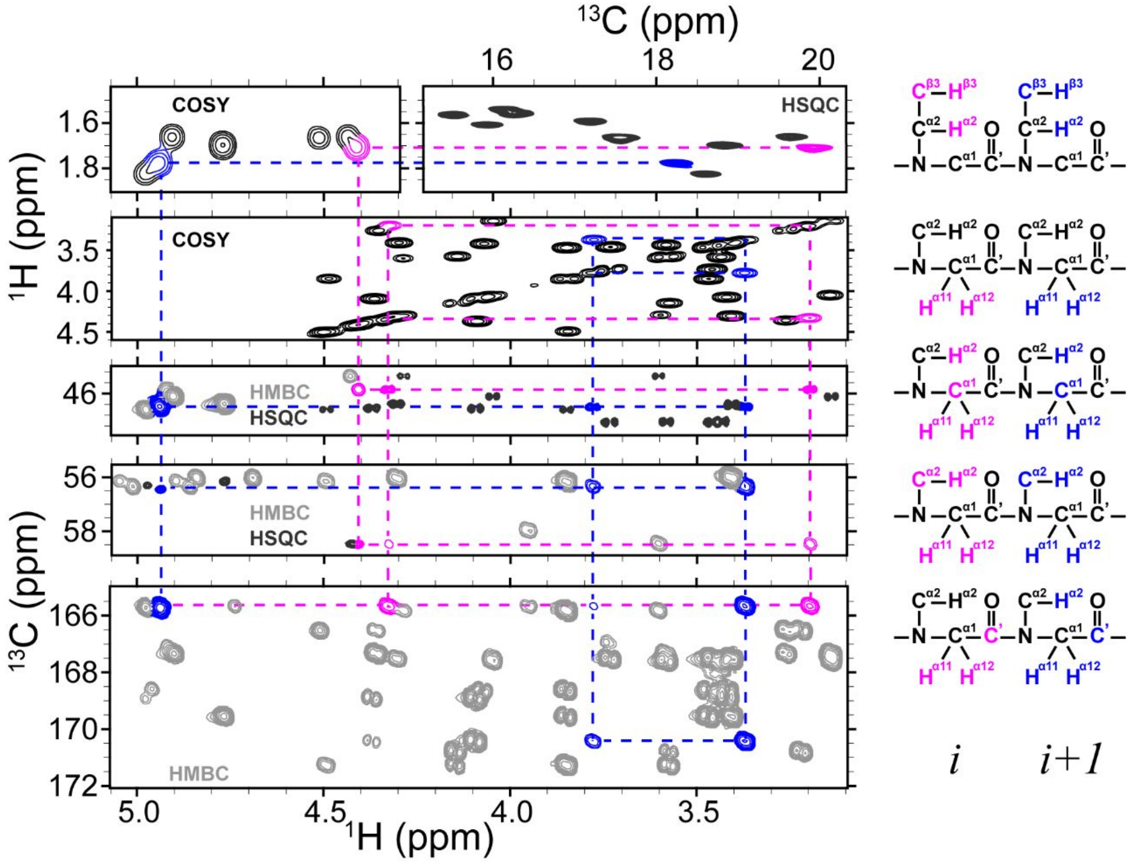

Figure S3. Sequential assignment of peptoid residues using 2D NMR spectroscopy.

Stepwise NMR assignment process using ^{1}H–^{1}H COSY, ^{1}H–^{13}C HSQC, and ^{1}H–^{13}C HMBC spectra, demonstrated with a *N*spe$_7$ peptoid spectra. **[Top panels]** One-bond ^{1}H–^{13}C correlations of methyl groups are identified from the HSQC spectrum. **[2nd panel]** Two-bond ^{1}H–^{1}H correlations of methyl and methylene groups are extracted from the COSY spectrum, guided by their chemical shifts. **[3rd and 4th panels]** Intra-residue correlations are established using HMBC, including methylene hydrogen–α-carbon ($H^{\alpha2}$–$C^{\alpha2}$) and backbone α-hydrogen–methylene carbon ($H^{\alpha2}$–$C^{\beta3}$) correlations. One-bond ^{1}H–^{13}C correlations of methylene and backbone α-carbon groups are also identified in HSQC. Additionally, two-bond ^{1}H–^{1}H correlations of backbone α-hydrogens are observed in the COSY spectrum (2nd panel). **[Bottom panel]** Sequential assignments are determined by detecting three-bond C'(*i*)–$H^{\alpha1}$(*i+1*) correlations in HMBC, establishing residue connectivity. These steps are systematically repeated to extend assignments along the sequence. Color-coded dashed lines (magenta and blue) indicate sequential correlations between residues. The right panel presents a schematic representation of key NMR correlations used for backbone and side-chain assignments.

(2) ^{1}H–^{13}C HSQC spectra

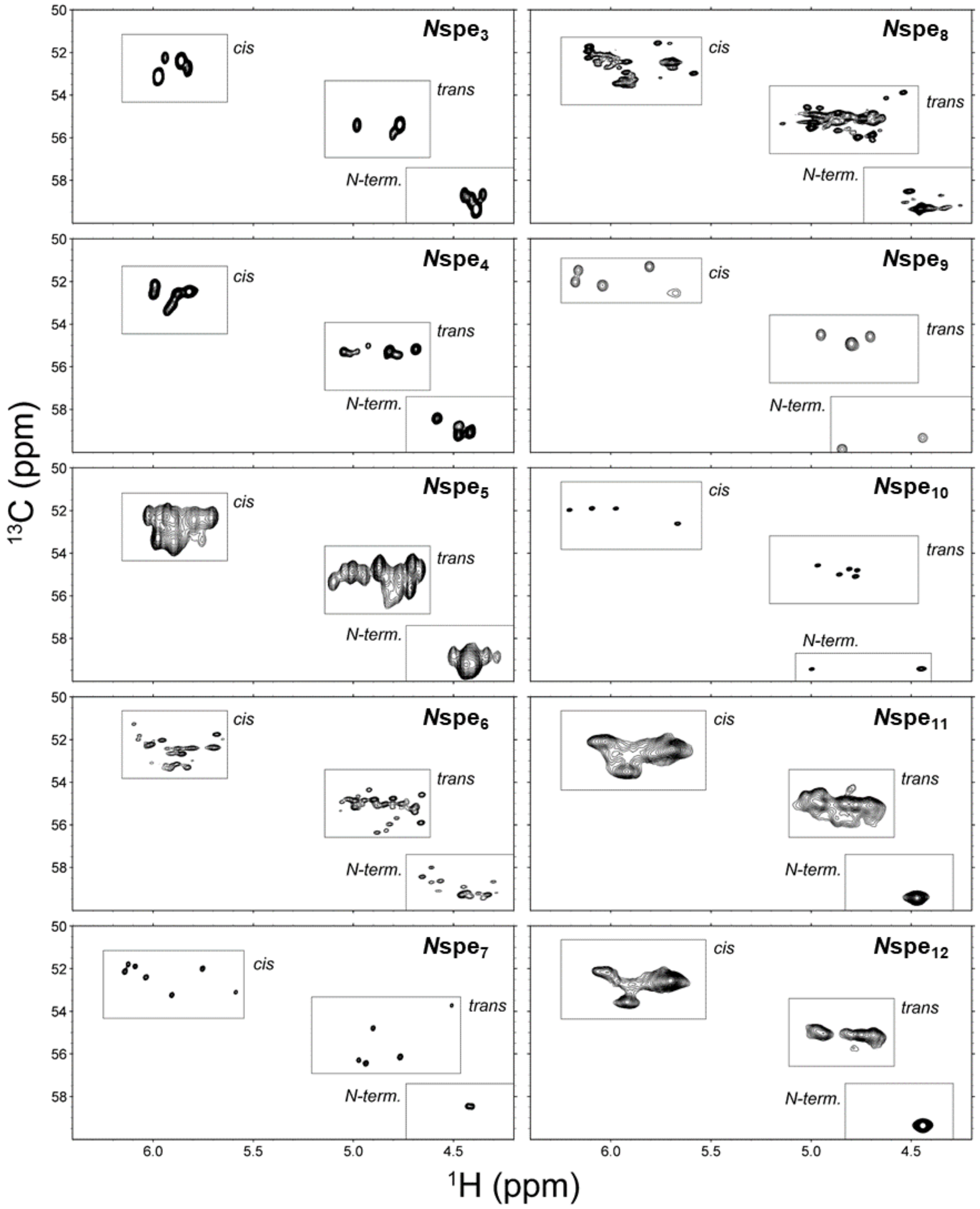

Figure S4. $C^{\alpha 2}$-$H^{\alpha 2}$ peaks from peptoids in ^{1}H–^{13}C HSQC spectra.

The *cis*, *trans*, and *N*-terminus regions are distinguished by boxes. The *cis* and *trans* conformations are differentiated based on their characteristic ^{13}C and ^{1}H chemical shifts, reflecting differences in backbone dihedral angles, and according NOE correlations. These spectral features provide insights into the conformational preferences of *N*spe peptoids as a function of sequence length.

(3) 2D NMR spectra of Nspe oligomers (Nspe$_4$ to Nspe$_{12}$)

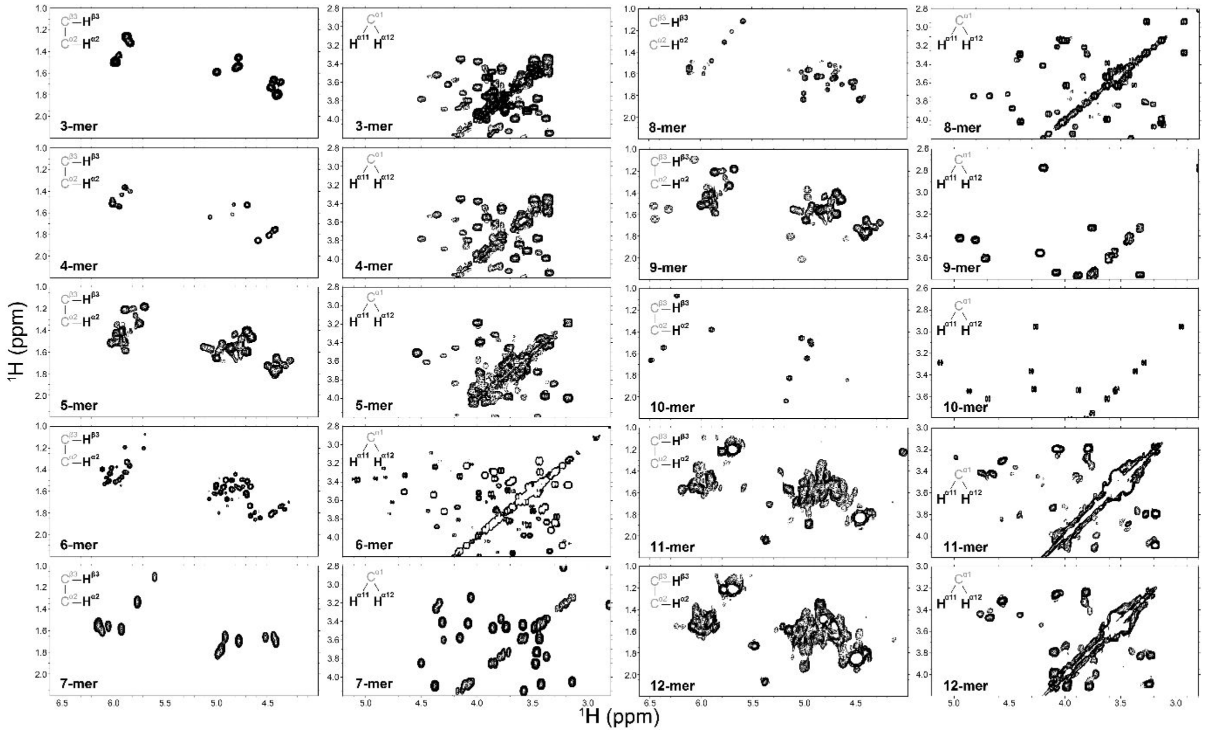

Figure S5. Expanded ^{1}H–^{1}H COSY spectra of *N*spe$_4$, *N*spe$_5$, *N*spe$_6$, *N*spe$_8$, *N*spe$_9$, *N*spe$_{10}$, *N*spe$_{11}$ and *N*spe$_{12}$ in $CDCl_3$ at 25 °C, recorded at a sample concentration of 50 mM.

Peaks corresponding to *N*-terminal residues were excluded from the calculation of $K_{cis/trans}$ (Figure 1B), as these termini were not capped with acyl group.

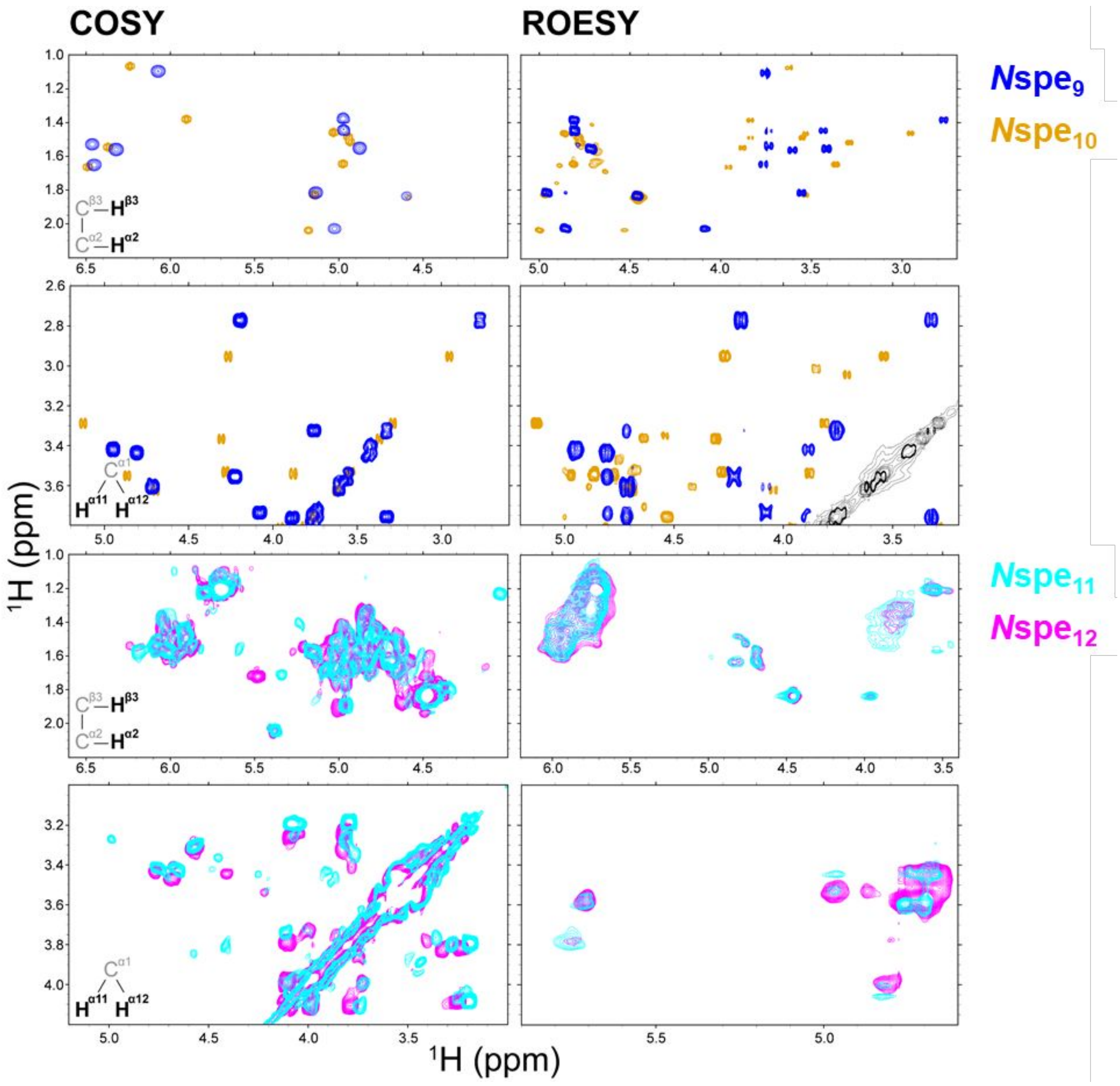

Figure S6. Comparison of COSY and ROESY Spectra for *N*spe$_9$ and *N*spe$_{10}$, *N*spe$_{11}$ and *N*spe$_{12}$ peptoids.

^{1}H–^{1}H COSY and ^{1}H–^{1}H ROESY spectra of the *N*spe$_9$ (blue), *N*spe$_{10}$ (orange), *N*spe$_{11}$ (cyan), and *N*spe$_{12}$ (magenta) peptoids. The *N*spe$_9$ and *N*spe$_{10}$ spectra exhibit minimal differences, while the *N*spe$_{11}$ and *N*spe$_{12}$ spectra show no significant variation between the two sequences.

Notably, the ^{1}H–^{1}H COSY and ^{1}H–^{13}C HSQC pattern of *N*spe$_9$ in $CDCl_3$ closely resembles that of the previously reported structure in acetonitrile, suggesting that the threaded-loop conformation is preserved across both solvent environments.

(4) Terminal protons

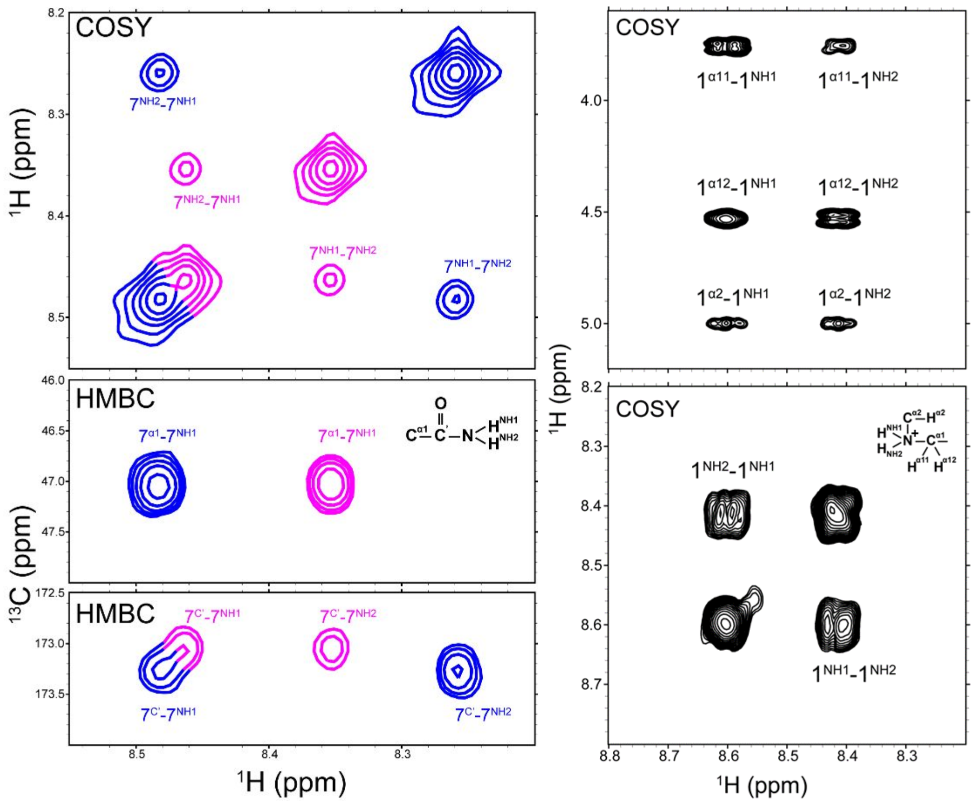

Figure S7. Assignment of the *C*-terminal amide (–$CONH_2$) resonances of the *N*spe_7 (left; magenta: Conformation 1, blue: Conformation 2) and the *N*-terminal amine (–NH_2^+) resonances of the *N*spe_{10} (right) by 2D NMR spectroscopy.

(5) Chemical shift of Nspe$_3$, Nspe$_7$, Nspe$_9$ and Nspe$_{10}$

Table S1. Chemical shifts of *N*spe$_3$ at 900 MHz in $CDCl_3$.

	#	C$^{\alpha1}$ (ppm)	H$^{\alpha11}$ (ppm)	H$^{\alpha12}$ (ppm)	$^2J_{HH}$ (Hz)	C$^{\alpha2}$ (ppm)	H$^{\alpha2}$ (ppm)	C$^{\beta3}$ (ppm)	H$^{\beta3}$ (ppm)	C' (ppm)
**Nspe$_3$ (cis,cis)**	1	45.80	3.745	3.877	15.27	59.39	4.394	19.71	1.804	167.1
	2	44.77	3.698	3.844	14.41	53.18	5.964	15.75	1.500	168.6
	3	44.73	3.45	3.508	17.84	52.42	5.857	15.4	1.262	171.4
**Nspe$_3$ (cis,trans)**	1	46.02	3.752	3.927	-	59.01	4.421	19.12	1.668	167.2
	2	44.44	3.831	4.120	19.17	52.81	5.824	15.87	1.320	166.1
	3	46.69	3.483	3.862	17.11	55.35	4.771	17.93	1.461	171.5
**Nspe$_3$ (trans,cis)**	1	45.58	3.834	3.834	58.71	58.71	4.45	19.78	1.738	168.3
	2	44.54	3.876	4.165	18.92	55.58	4.772	18.78	1.535	168.0
	3	45.90	3.650	16.98	52.28	52.28	5.936	15.89	1.436	171.5
**Nspe$_3$ (trans,trans)**	1	45.61	3.913	3.986	14.47	58.69	4.361	19.56	1.684	166.0
	2	45.74	3.619	4.257	18.12	55.95	4.798	18.30	1.560	168.0
	3	46.49	3.543	3.884	18.38	55.45	4.979	18.25	1.593	171.7

Table S2. Chemical shifts of *N*spe$_7$ at 900 MHz in $CDCl_3$.

	#	C$^{\alpha1}$ (ppm)	H$^{\alpha11}$ (ppm)	H$^{\alpha12}$ (ppm)	$^2J_{HH}$ (Hz)	C$^{\alpha2}$ (ppm)	H$^{\alpha2}$ (ppm)	C$^{\beta3}$ (ppm)	H$^{\beta3}$ (ppm)	C' (ppm)	H^{CONH1} (ppm)	H^{CONH2} (ppm)
**Nspe$_7$ Conformation 1**	**1**	45.85	3.196	4.326	15.53	58.49	4.408	19.93	1.706	165.7		
	**2**	46.49	3.371	3.777	17.34	56.45	4.937	18.23	1.773	170.4		
	**3**	46.55	4.093	4.371	20.50	53.23	5.907	17.18	1.591	168.9		
	**4**	44.62	3.419	4.073	21.19	51.99	5.752	15.83	1.338	168.6		
	**5**	43.08	3.468	3.851	20.78	52.40	6.037	15.50	1.563	169.6		
	**6**	46.39	3.407	4.303	18.49	56.15	4.767	18.83	1.695	167.6		
	**7**	47.06	3.458	3.733	21.90	52.15	6.142	16.30	1.551	173.3	8.258	8.481
**Nspe$_7$ Conformation 2**	**1**	45.35	3.602	4.285	17.88	58.45	4.428	19.66	1.658	165.8		
	**2**	46.59	3.849	4.491	18.43	56.29	4.973	18.59	1.822	171.3		
	**3**	44.56	3.575	4.147	19.70	51.89	6.090	15.92	1.605	170.8		
	**4**	44.03	2.810	3.220	20.22	53.12	5.587	16.07	1.106	166.5		
	**5**	43.46	3.260	4.360	18.80	53.72	4.509	17.54	1.663	167.4		
	**6**	46.11	3.139	4.048	18.25	54.78	4.901	17.56	1.663	167.6		
	**7**	47.04	3.435	3.582	19.45	51.79	6.126	16.13	1.546	173.1	8.353	8.465

Table S3. Chemical shifts of *N*spe$_9$ and *N*spe$_{10}$ at 900 MHz in $CDCl_3$.

	#	$C^{\alpha 1}$ (ppm)	$H^{\alpha 11}$ (ppm)	$H^{\alpha 12}$ (ppm)	$^2J_{HH}$ (Hz)	$C^{\alpha 2}$ (ppm)	$H^{\alpha 2}$ (ppm)	$C^{\beta 3}$ (ppm)	$H^{\beta 3}$ (ppm)	C' (ppm)	H^{NH1} (ppm)	H^{NH2} (ppm)
*N*spe$_9$ Conformation 1	**1**	49.51	4.127	41.94	59.88	4.851	19.76	2.018	168.3			
	**2**	46.1	3.602	4.710	22.41	52.19	6.048	17.47	1.558	169.5		
	**3**	45.37	3.734	4.078	20.40	52.03	6.184	15.42	1.531	168.8		
	**4**	45.01	3.434	4.812	18.61	54.97	4.812	19.22	1.444	167.4		
	**5**	45.01	3.548	4.224	18.93	54.50	4.958	19.18	1.808	167.7		
	**6**	44.95	2.764	4.192	18.58	54.90	4.790	17.97	1.379	167.4		
	**7**	44.14	3.316	3.749	20.81	51.30	5.814	15.69	1.093	167.0		
	**8**	45.6	3.422	4.944	19.35	54.58	4.712	18.45	1.549	168.1		
	**9**	44.67	3.772	3.897	19.01	51.47	6.169	16.02	1.643	172.2	8.098	8.608
*N*spe$_9$ Conformation 2	**1'**					59.35	4.450	19.38	1.829			
*N*spe$_{10}$ Conformation 1	**1**	46.02	3.757	4.529	24.81	59.38	4.998	18.39	2.031	168.0		
	**2**	46.03	3.622	4.694	21.21	51.89	5.976	17.17	1.075	170.5		
	**3**	45.38	3.836	4.042	19.61	52.60	5.668	15.18	1.381	167.9		
	**4**	45.57	3.551	4.862	18.41	55.07	4.783	19.30	1.484	166.8		
	**5**	44.37	3.532	4.280	18.41	54.55	4.969	18.72	1.824	168.1		
	**6**	44.52	2.953	4.263	18.81	54.97	4.862	17.70	1.458	167.5		
	**7**	44.28	3.540	3.878	20.41	51.90	6.096	15.70	1.545	169.1		
	**8**	45.05	3.289	5.124	18.81	54.79	4.772	17.82	1.516	167.4		
	**9**	44.34	3.812	3.956	18.81	51.97	6.209	15.83	1.661	168.4		
	**10**	45.42	3.366	4.305	19.21	54.68	4.810	17.57	1.642	169.7	8.416	8.601
*N*spe$_{10}$ Conformation 2	**1'**					59.37	4.450	19.27	1.842			

(6) Conformational analysis of Nspe₃

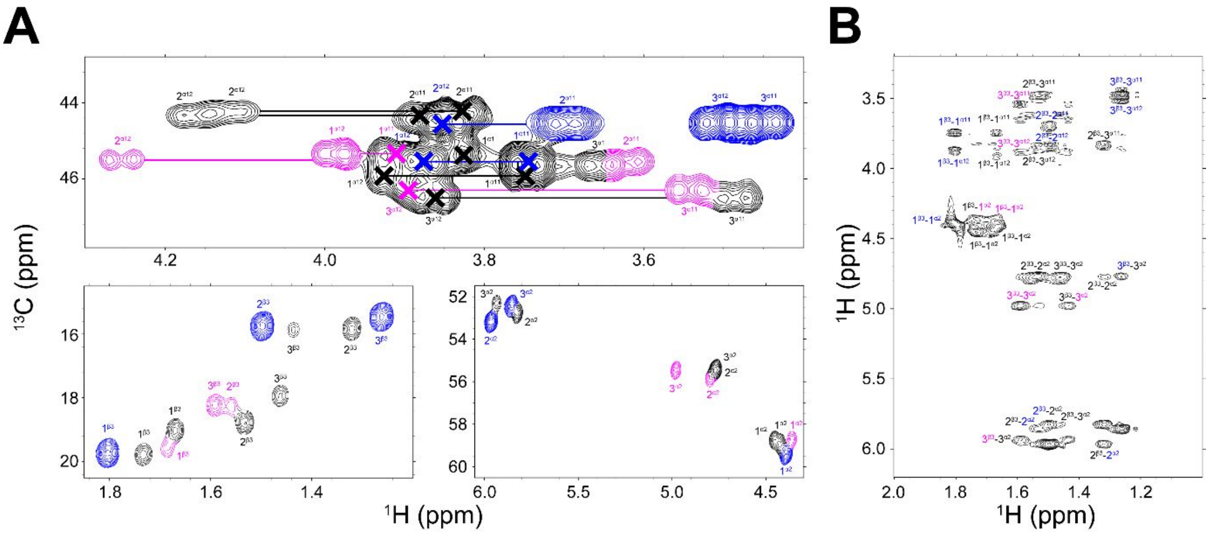

Figure S8. NMR-based conformational analysis of *N*spe$_3$ (**1**). Expanded plots of (A) ^{1}H–^{13}C HSQC spectra and (B) ^{1}H–^{1}H ROESY in $CDCl_3$ at 25 °C with a sample concentration of 50 mM.

All four theoretically possible conformations were observed through 2D NMR spectroscopy. However, assignment of the *trans*-amide in the *ct* (*cis*, *trans*) conformer (Conformation 2) and the *cis*-amide in the *tc* (*trans*, *cis*) conformer (Conformation 3) was complicated due to peak overlap in the ^{1}H–^{13}C HSQC and ^{1}H–^{1}H ROESY spectra (Figure S8). To resolve these ambiguities, DFT calculations were employed to predict chemical shifts and validate the *cis*- and *trans*-amide assignments for each conformer (Figure S9 and S10).

Table S4. *N*spe$_3$ population table

	#	COSY H$^{\alpha2}$-H$^{\beta3}$ Intensity	Population	Expected Conformation
	1	0.54		
***N*spe$_3$ Conformation 1**	2	0.64	54%	*cc*
	3	0.45		
	1	0.19		
***N*spe$_3$ Conformation 2**	2	0.22	19%	*c*(*t*)*
	3	0.14		
	1	0.18		
***N*spe$_3$ Conformation 3**	2	0.20	15%	*t*(*c*)*
	3	0.08		
	1	0.08		
***N*spe$_3$ Conformation 4**	2	0.10	12%	*tt*
	3	0.17		

*Further clarified through DFT calculations (Figure S9 and S10)

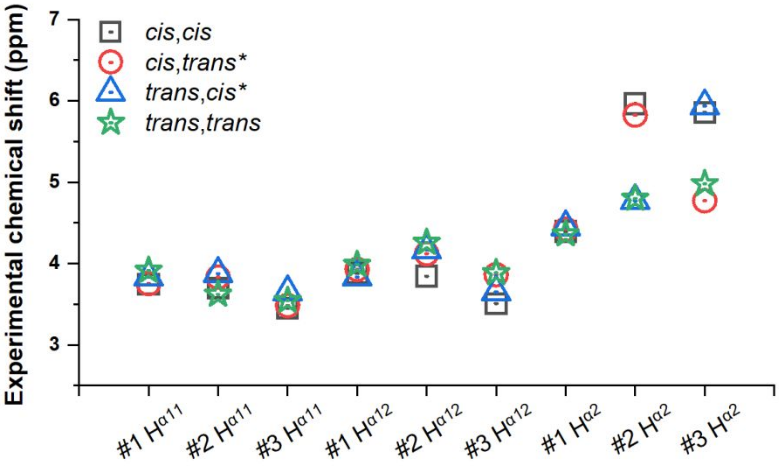

Figure S9. Experimental ^{1}H NMR chemical shifts of the $H^{\alpha 11}$, $H^{\alpha 12}$, and $H^{\alpha 2}$ for the four *N*spe$_3$ isomers.

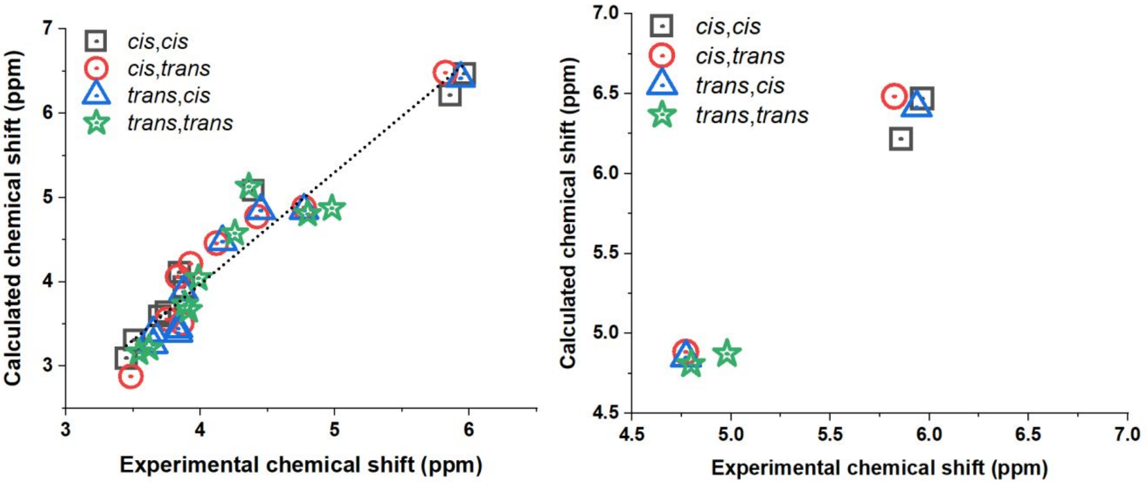

Figure S10. Correlation between calculated and experimental chemical shifts for all targeted protons, with the dotted line showing the linear fit (y=1.33x-1.34, R^2=0.924) (left panel). (B) Correlation plot focusing on the $H^{\alpha 2}$ (right panel).

Figure S9 displays the ^{1}H NMR chemical shifts for the $H^{\alpha 11}$, $H^{\alpha 12}$, and $H^{\alpha 2}$ of the three residues. While the chemical shifts of other protons differ by approximately 0.5 ppm, the $H^{\alpha 2}$ of the second and third peptoid monomers exhibit a more substantial difference of ~1.0 ppm. As this variation showed a clear dependence on the *cis*/*trans* conformation, our comparative analysis was focused on these specific protons. Figure S10A presents both the experimental and calculated chemical shifts for the target protons ($H^{\alpha 11}$, $H^{\alpha 12}$, and $H^{\alpha 2}$). The mean of absolute error (MAE) is 0.30 ppm for these protons.[11] In Figure S10B, which focuses on the α_2 protons of the second and third peptoid monomers, it is confirmed that the trend of the chemical shift is dependent on the *cis*/*trans* conformation with agreement between the experimental and calculated values.

(7) DMSO titration

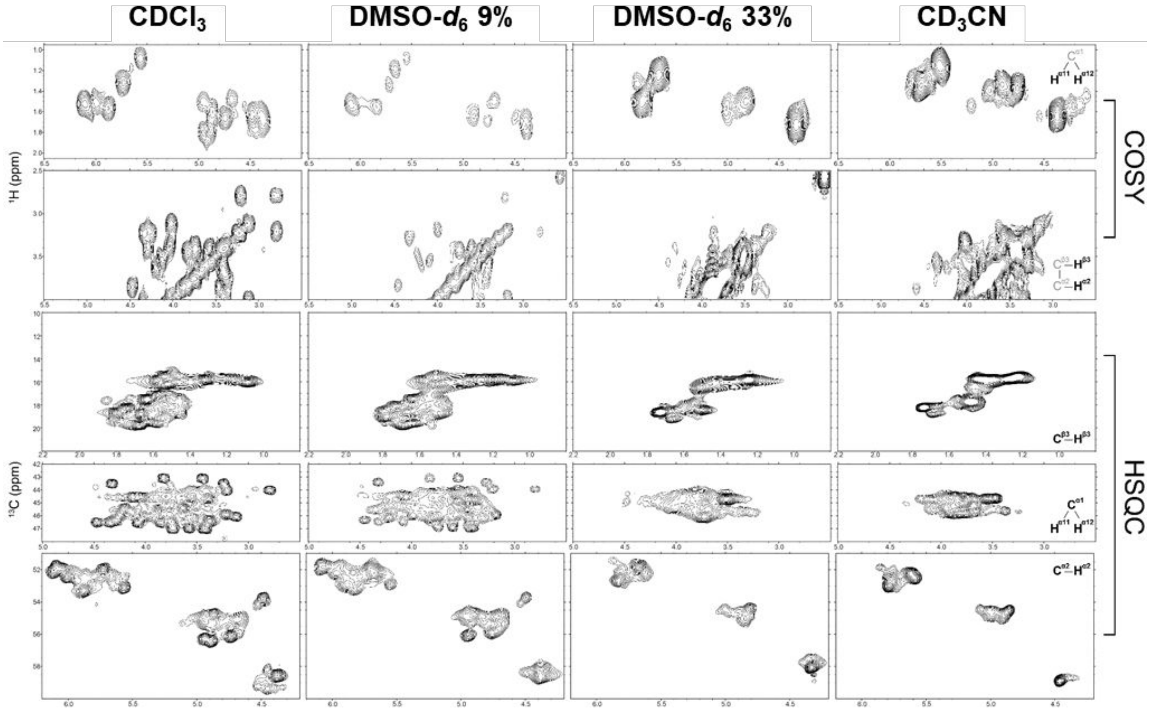

Figure S11. Effect of DMSO-d_6 addition on the ^{1}H–^{1}H COSY (top) and ^{1}H–^{13}C HSQC (bottom) spectra of the *N*spe$_7$ at 20 °C. Overlay of COSY spectra recorded at 600 MHz in $CDCl_3$ containing 0%, 9%, 33% DMSO-d_6, and CD_3CN.

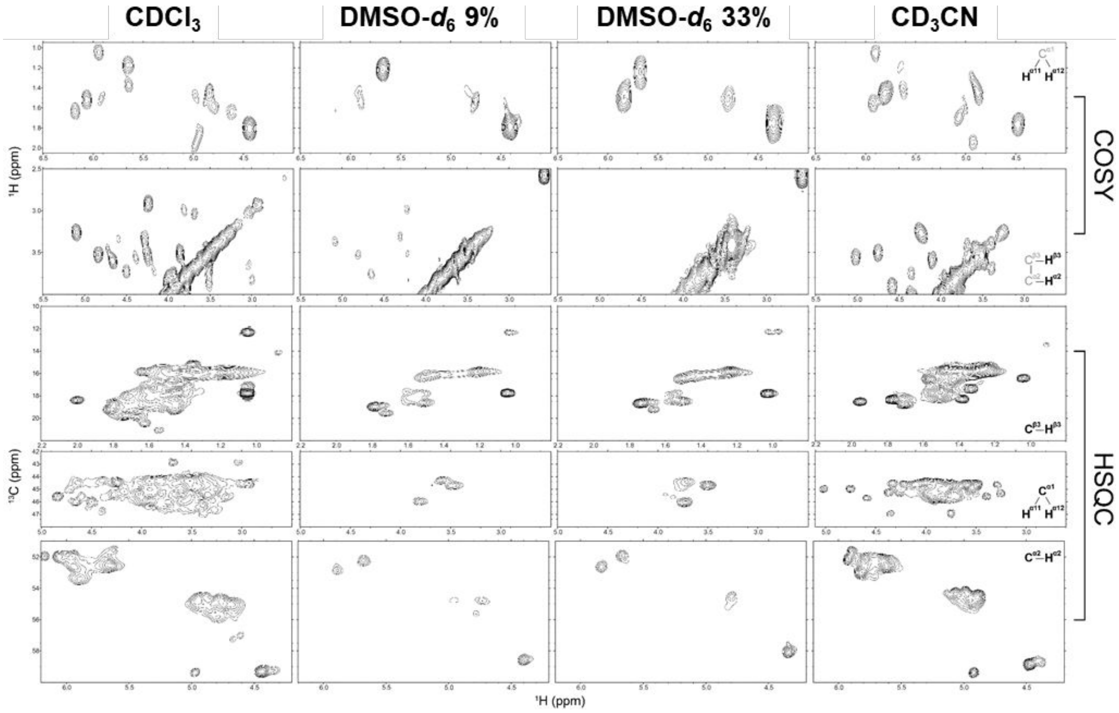

Figure S12. Effect of DMSO-d_6 addition on the ^{1}H–^{1}H COSY (top) and ^{1}H–^{13}C HSQC (bottom) spectra of the *N*spe$_{10}$ at 20 °C. Overlay of COSY spectra recorded at 600 MHz in $CDCl_3$ containing 0%, 9%, and 33% DMSO-d_6.

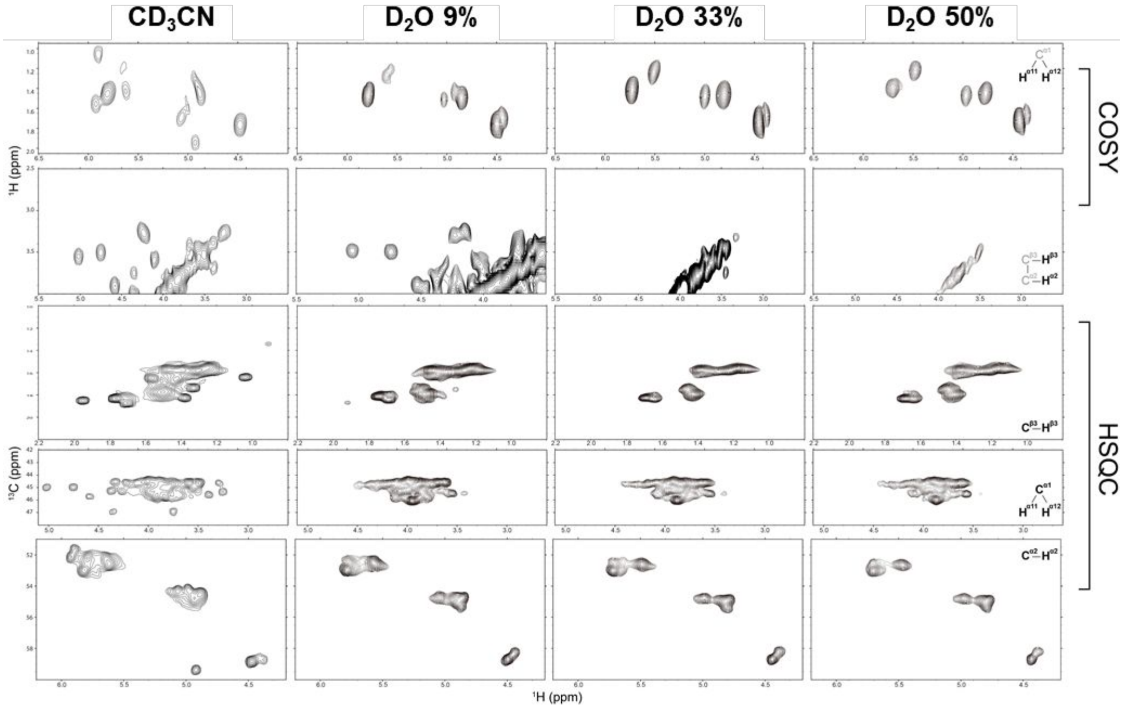

Figure S13. Effect of D_2O addition on the ^1H–^1H COSY (top) and ^1H–^{13}C HSQC (bottom) spectra of the *N*spe$_{10}$ at 20 °C. Overlay of COSY spectra recorded at 600 MHz in CD_3CN containing 9%, 33%, 50% D_2O, and CD_3CN.

For *N*spe$_{10}$, DMSO-d_6 titration similarly disrupted the closed fold; however, a population of the closed conformer persisted in CD_3CN. Further titration with D_2O into the CD_3CN solution was required to shift the equilibrium toward the helical state, indicating that the intramolecular hydrogen bonds in *N*spe$_{10}$ are more stable in acetonitrile than those in *N*spe$_7$.

(8) Variable temperature (VT) NMR

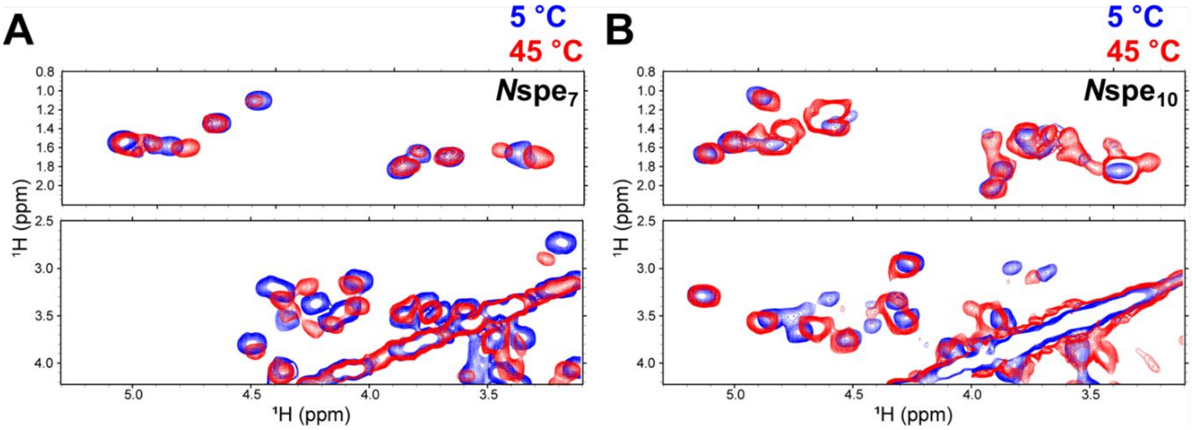

Figure S14. Temperature-dependent ^{1}H–^{1}H COSY spectra of the *N*spe$_7$ and the *N*spe$_{10}$ at 5 °C (blue) and 45 °C (red). Overlay of COSY spectra recorded at 600 MHz in $CDCl_3$. Spectra at 5 °C are shown in blue, and those at 45 °C in red, illustrating temperature-induced chemical-shift changes and line-width variations. Contour levels in each spectrum increase successively by factors of two.

(9) Competing interactions drive solvent-dependent folding

This contrast can be rationalized by the distinct balance of intramolecular noncovalent interactions across solvent environments. In polar solvents such as water and acetonitrile, intramolecular hydrogen bonding between the chain termini is suppressed by competitive solvation, and folding is governed primarily by the steric interactions from the bulky Nα-chiral aromatic side chains and $n\pi^*$ electronic interactions between the aromatic ring and the preceding carbonyl group. These effects that collectively enforce cis-amide and drive PPI-like helical conformations. In chloroform, the absence of competitive solvation frees the protonated *N*-terminal amine and *C*-terminal primary amide to engage in strong end-to-end intramolecular hydrogen bonds. Although the steric and $n\pi^*$ interactions that favor *cis*-amide and helical conformation remain present, the emergence of terminal hydrogen bonds introduce a competing driving force that shifts the equilibrium toward closed, loop-type conformations. Importantly, this conformational preference is not uniformly accessible across all chain lengths; rather, it is specifically favored at lengths that provide the appropriate end-to-end distance and geometry for hydrogen bonding—most notably *N*spe$_7$, *N*spe$_9$, and *N*spe$_{10}$—as evidenced by NMR and MD analyses.

The observation of loop structures over helices in chloroform suggests that end-to-end hydrogen bonding provides stronger thermodynamic stabilization than the helix-promoting steric and $n\pi^*$ effects, cooperatively driving *N*spe oligomers toward closed conformations in the nonpolar environment–and implying that peptoids may similarly adopt stabilized structures in lipid-like environments by masking polar functional groups.

5. Polar surface area (PSA) and permeability implications

Table S5. Topological and conformational PSA calculation of *N*spe$_7$ and *N*spe$_{10}$.

Compound	Total PSA[*] (topological, Å^2)	3D polar solvent-accessible surface area (SASA)[*] (conformational, Å^2)
*N*spe$_{7,\ \mathrm{Conformation\ 1}}$	181.56	41.55
*N*spe$_{7,\ \mathrm{Conformation\ 2}}$	181.56	79.88
*N*spe$_{10}$	242.49	83.07

[*]Calculated with Discovery Studio v.17.1

Such structural homogeneity in chloroform demonstrates that peptoid sequences can achieve distinct, well-defined secondary structures stabilized by intramolecular hydrogen bonding and optimal chain length under lipid-like conditions. Importantly, these closed conformations allow the foldamer to mask polar functional groups from solvent, which in turn promotes passive membrane permeability. One way to quantify this effect is calculating polar solvent accessible surface area (SASA) and polar surface area (PSA) from MD-derived conformational ensembles. Computationally derived fractional PSA (fPSA = polar SASA / total SASA) values support the potential for passive membrane permeability, with values of 0.143 for *N*spe$_7$ and 0.135 for *N*spe$_{10}$. Both fall within the empirical range displaying favorable membrane permeability.[16,17] Energy-weighted effective PSA (ePSA) was calculated from MD ensembles were 54.1 Å^2 for *N*spe$_7$ and 83.1 Å^2 for *N*spe$_{10}$ (Table S5). Compared to topological total PSA (TPSA) values of 181.6 Å^2 and 242.5 Å^2, respectively, folding into closed structure resulted effective reduction of PSA in chloroform environment. The more reduction of solvent accessible area of *N*spe$_{10}$ likely reflects more extensive intramolecular masking of polar groups by bulky side chains, further implying a presence of chameleonic character.[16,18]

Table S6. PAMPA permeability of *N*spe$_7$, *N*spe$_9$, and *N*spe$_{10}$.

Compound	Permeability ($\times 10^{-6}$ cm/s)[a]	R (%)[b]
Cz	8.6 ± 0.6	96 ± 0.6
CsA	1.4 ± 0.2	77 ± 4
*N*spe$_7$	0.02 ± 0.01	25 ± 2
*N*spe$_9$	< 0.005[*]	-
*N*spe$_{10}$	ND[*]	-

[*]MS signal was inherently low. [a]Permeability was determined by PAMPA. [b]R (%): mass retention. CsA, cyclosporin A. Cz, carbamazepine.

The closed conformations of *N*spe$_7$ and *N*spe$_{10}$ are stabilized by end-to-end intramolecular hydrogen bonding and hydrophobic side-chain shielding, which together bury the polar backbone amides and reduce the solvent-exposed polar surface in the low-dielectric environment (Table S5). This behavior

is the defining feature of chameleonic molecules, which switch between an open, polar-solvated conformation in aqueous or polar media and a closed, polarity-masked conformation in nonpolar or membrane-like environments.

Cyclosporin A is a representative chameleonic macrocycle: in low-dielectric media it forms intramolecular hydrogen bonds that shield its polar groups, lowering its effective polarity and enabling passive membrane permeability. The closed folds of *N*spe$_7$ and *N*spe$_{10}$ employ the same polarity-masking strategy—intramolecular hydrogen bonding combined with hydrophobic side-chain shielding—and can therefore be regarded as peptoid analogues of this macrocyclic behavior.

Consistent with this picture, *N*spe$_7$ showed only marginal passive permeability in the PAMPA assay (Table S6). This may reflect a large free-energy barrier (ΔG) between the open and closed conformations that slows the interconversion required for efficient permeation. Moreover, unlike conformationally pre-organized macrocycles such as cyclosporin A, a linear peptoid must pay a greater entropic and enthalpic cost to access the closed, polarity-masked state, which may further attenuate permeation. A quantitative evaluation of these conformational energetics lies beyond the scope of the present study. However, future studies are warranted to explore strategies for lowering the free-energy barrier associated with conformational switching, particularly through covalent-linkage-based cyclization approaches.

6. MD simulation

(1) Overview

We used a two-stage molecular simulation approach to determine solution structures of H-(*N*spe)$_7$-NH$_2$ and H-(*N*spe)$_{10}$-NH$_2$ (*N*spe$_{10}$) in chloroform based on the NOE cross-peaks seen in the 2D NMR spectroscopy. The H-(*N*spe)$_7$-NH$_2$ molecule showed *two* sets of peaks in slow exchange, indicating two competing stable structures (which we call *N*spe$_{7\text{-}1}$ and *N*spe$_{7\text{-}2}$). Separate structural refinements were performed for each set of cross-peaks.

In the first stage, we performed Hamiltonian replica exchange (HREX) with flat-bottom distance restraints and scaled backbone amide torsion barriers. This procedure generated conformations with correct backbone amide isomers that were mostly consistent with the distance restraints, but undersampled backbone phi-angle and side-chain chi-angle degrees of freedom.

In the second stage, we used temperature replica exchange molecular dynamics (REMD) simulations without restraints to thoroughly sample the remaining degrees of freedom. We then used the Bayesian Inference of Conformational Populations (BICePs) algorithm to reweight the simulated populations in agreement with the experimental distance restraints for each molecule (*N*spe$_{7\text{-}1}$, *N*spe$_{7\text{-}2}$ and *N*spe$_{10}$). BICePs is a maximum-entropy approach to infer state populations most consistent with ensemble-

averaged measurements, using a Bayesian framework to infer experimental and model uncertainties directly from the data. For each structural refinement, the result is a conformational ensemble dominated by a small number of backbone torsion states. We selected ten exemplar structures from the most populated states to assemble an ensemble of ~ 30 structures representative of the folded state.

(2) Determination of NOE distance restraints

Theoretical interproton distances were determined from the NOE cross-peaks using the formula $r = (1.8\ \text{Å})|V_{ref}/V|^{1/6}$, where V is the integrated cross-peak volume for the proton pair, and V_{ref} is the cross-peak volume for a reference pair of protons. For this reference, we chose the geminal protons on the alpha carbon of the *C*-terminal *N*spe residue. Based on the calculated theoretical distance, we assigned the qualitative categories: strong ($r < 2.5$ Å), medium (2.5 Å $\leq r \leq$ 3.5 Å), and weak (3.5 Å $<$ r $\leq$ 5 Å). Integrated peak volumes and the computed distance categories of interproton pairs for $N\text{spe}_{7\text{-}1}$ and $N\text{spe}_{7\text{-}2}$ are shown in Table S7 and shown for $N\text{spe}_{10}$ in Table S8.

Table S7. Computed distance categories of interproton pairs for *N*spe$_{7\text{-}1}$, *N*spe$_{7\text{-}2}$.

*N*spe$_{7\text{-}1}$		*N*spe$_{7\text{-}2}$	
Interproton pair	Distance category	Interproton pair	Distance category
$1^{\alpha11}$-$2^{\alpha2}$	strong	$1^{\alpha12}$-$2^{\alpha2}$	strong
$1^{\alpha12}$-$2^{\alpha2}$	strong	$2^{\alpha12}$-$3^{\alpha12}$	strong
$3^{\alpha12}$-$2^{\alpha12}$	strong	$5^{\alpha11}$-$4^{\alpha12}$	strong
$4^{\alpha11}$-$3^{\alpha11}$	strong	$5\alpha11$-$6^{\alpha2}$	strong
$4^{\alpha11}$-$5^{\alpha2}$	strong	$5\alpha12$-$6^{\alpha2}$	strong
$4^{\alpha12}$-$5^{\alpha2}$	strong	$6^{\alpha11}$-$7^{\alpha12}$	strong
$5^{\alpha11}$-$6^{\alpha2}$	strong	$7^{\alpha12}$-$6^{\alpha12}$	strong
$5^{\alpha12}$-$6^{\alpha2}$	strong	$2^{\beta3\text{-}\alpha11}$	strong
$6^{\beta3}$-$5^{\alpha11}$	strong	$3^{\beta3\text{-}\alpha11}$	strong
$6^{\alpha11}$-$7^{\alpha12}$	strong	$4^{\beta3\text{-}\alpha11}$	strong
$7^{\alpha12}$-$6^{\alpha12}$	strong	$5^{\beta3\text{-}\alpha11}$	strong
$2^{\beta3\text{-}\alpha11}$	strong	$6^{\beta3\text{-}\alpha11}$	strong
$3^{\beta3\text{-}\alpha11}$	strong	$6^{\alpha12}$-7^{NH1}	medium
$6^{\beta3\text{-}\alpha11}$	strong	$1^{\alpha11}$-$2^{\alpha2}$	medium
$7^{\beta3\text{-}\alpha11}$	strong	$2^{\beta3}$-$1^{\alpha11}$	medium
$6^{\alpha12}$-7^{NH2}	medium	$4^{\alpha12}$-$3^{\alpha12}$	medium
$5^{\beta3}$-$4^{\alpha11}$	medium	$6^{\beta3}$-$5^{\alpha12}$	medium
$2^{\alpha11\text{-}\alpha2}$	medium	$1^{\alpha11\text{-}\alpha2}$	medium
$3^{\alpha11\text{-}\alpha2}$	medium	$2^{\alpha11\text{-}\alpha2}$	medium
$7^{\alpha11\text{-}\alpha2}$	medium	$3^{\alpha11\text{-}\alpha2}$	medium
$6^{\alpha12}$-7^{NH1}	weak	$4^{\alpha11\text{-}\alpha2}$	medium
$3^{\alpha12}$-7^{NH1}	weak	$5^{\beta3\text{-}\alpha12}$	medium
$7^{\alpha12\text{-}NH2}$	weak	$5^{\alpha12\text{-}\alpha2}$	medium
$7^{\alpha12\text{-}NH1}$	weak	$6^{\alpha12}$-7^{NH2}	weak
		$3^{\alpha12}$-7^{NH1}	weak
		$3^{\alpha12}$-7^{NH2}	weak
		$3^{\alpha11}$-7^{NH1}	weak
		$7^{\alpha11\text{-}\alpha2}$	weak
		$7^{\alpha11\text{-}NH1}$	weak
		$7^{\alpha11\text{-}NH2}$	weak

Table S8. Computed distance categories of interproton pairs for *N*spe$_{10}$.

*N*spe$_{10}$			
Interproton pair	Distance category	Interproton pair	Distance category
$10^{\alpha11}$-$1^{\alpha12}$	strong	$9^{\beta3\text{-}\alpha12}$	strong
$2^{\alpha11}$-$3^{\alpha12}$	strong	$10^{\beta3\text{-}\alpha11}$	strong
$3^{\alpha11}$-$4^{\alpha2}$	strong	$10^{\alpha11\text{-}\alpha2}$	strong
$3^{\alpha12}$-$4^{\alpha2}$	strong	$5^{\alpha11}$-$1^{\alpha2}$	medium
$4^{\alpha11}$-$5^{\alpha2}$	strong	$7^{\beta3}$-$1^{\beta3}$	medium
$6^{\beta3}$-$5^{\alpha11}$	strong	$5^{\beta3}$-$1^{\beta3}$	medium
$5^{\alpha12}$-$6^{\alpha2}$	strong	$3^{\beta3}$-$1^{\alpha11}$	medium
$6^{\alpha11}$-$7^{\alpha11}$	strong	$8^{\alpha12}$-1^{NH+1}	medium
$7^{\alpha11}$-$8^{\alpha2}$	strong	$8^{\alpha12}$-1^{NH+2}	medium
$7^{\alpha12}$-$8^{\alpha2}$	strong	$6^{\alpha12}$-1^{NH+2}	medium
$8^{\alpha11}$-$9^{\alpha11}$	strong	$1^{\alpha11}$-$2^{\alpha12}$	medium
$9^{\alpha11}$-$10^{\alpha2}$	strong	$3^{\alpha12}$-$2^{\alpha12}$	medium
$9^{\alpha12}$-$10^{\alpha2}$	strong	$4^{\beta3}$-$3^{\alpha11}$	medium
$1^{\beta3\text{\_}\alpha11}$	strong	$4^{\beta3}$-$3^{\alpha12}$	medium
$1^{\beta3\text{-}\alpha12}$	strong	$1^{\alpha2}$-1^{NH+1}	medium
$1^{\alpha11\text{-}\alpha2}$	strong	$1^{\alpha2}$-1^{NH+2}	medium
$2^{\beta3\text{-}\alpha11}$	strong	$1^{\beta3}$-1^{NH+2}	medium
$3^{\beta3\text{-}\alpha11}$	strong	$1^{\alpha12}$-1^{NH+1}	medium
$4^{\beta3\text{-}\alpha11}$	strong	$1^{\alpha12}$-1^{NH+2}	medium
$5^{\beta3\text{-}\alpha11}$	strong	$1^{\alpha11}$-1^{NH+1}	medium
$6^{\beta3\text{-}\alpha11}$	strong	$1^{\beta3}$-1^{NH+1}	medium
$7^{\beta3\text{-}\alpha12}$	strong	$6^{\alpha12}$-1^{NH+1}	weak
$8^{\beta3\text{-}\alpha11}$	strong	$1^{\alpha11}$-1^{NH+2}	weak

(3) Molecular Simulation

All simulations were performed on the Owlsnest high performance computing cluster at Temple University using GROMACS version 2021.2.[19] Polypeptoid topologies were modeled using the STEPs (Systematic and Extensible Force Field for Peptoids) force field.[20] STEPs is an AMBER-compatible force field with bonded terms reparametrized against DFT potential energy surfaces using GAFF2[21,22] as starting point, and RESP partial charges.

While the current release of STEPs provides topologies for 70 different sidechains including *N*spe, these topologies were designed for use in polypeptoids with capping groups. Since STEPs did not contain parameters for the uncapped, positively charged *N*-terminal residue +H-(*N*spe), we performed a custom parameterization of this residue using the STEPs protocol.[20]

Molecular topologies for chloroform solvent were generated using GAFF 2.1[23] and the AM1-BCC method for partial charges,[24] according to protocols described by Goold et al. (2025).[25] The $N\text{spe}_{7\text{-}1}$ simulation system consisted of one polypeptoid molecule (net charge +1), 398 solvent molecules, and a chloride counterion (charge -1) in a cubic period box of length 4.00035 nm. The $N\text{spe}_{7\text{-}2}$ simulation system consisted of one polypeptoid molecule (net charge +1), 396 solvent molecules, and a chloride counterion (charge -1) in a cubic period box of length 3.80717 nm. The $N\text{spe}_{10}$ simulation system consisted of 1 polypeptoid molecule (net charge +1), 609 solvent molecules, and a chloride counterion (charge -1) in a cubic period box of length 4.4045 nm.

Systems were steepest-descent minimized for 50000 steps, and then equilibrated at constant temperature and volume (NVT) for 100 ps at 300 K using a leap-frog integrator with 2 fs time step and a modified velocity-scaling Berendsen thermostat. Systems were then equilibrated at constant pressure and temperature (NPT) for 100 ps at 300 K and 1 bar, using a Berendsen barostat. Nonbonded cutoffs of 10 Å were used, and Particle Mesh Ewald (PME) was used to model electrostatics. The LINCS algorithm was used to constrain hydrogen bond lengths.[26]

(4) Hamiltonian Replica Exchange (HREX) simulations

In the HREX method, a number of trajectory replicas indexed by $i = 1, ..., N$, corresponding to different thermodynamic ensembles, are simulated in parallel. During the simulation, attempts are made periodically to exchange molecular configurations between two ensembles i and j according to the Metropolis criterion acceptance probability $P_{\text{acc}} = \min(1, \exp(\beta[U_i(x_i) - U_i(x_j) + U_j(x_j) - U_j(x_i)]))$, where $U_i(x)$ is the potential energy of a configuration x in thermodynamic ensemble i, x_i is a molecular configuration from ensemble i, and $\beta = 1/k_B T$ where k_B is Boltzmann's constant and T is the temperature.

The HREX simulations used a series of potential energy functions incorporating both flat-bottom distance restraints of various strengths and scaled omega-angle torsion potentials, defined as $U_i(x) = U^{(0)}(x) + \lambda_i U_{\text{rest}}(x) + \lambda_i' U_{\text{torsion}}(x)$. The potential $U_{\text{rest}}(x)$ (described in more detail below) enforces the experimental distances: the first replica ($i = 0$) has the restraint potential at full-strength ($\lambda_{i=0} = 1$), and each next replica reduces the strength of the potential until the last replica (i=15), which has the restraint potential fully turned off ($\lambda_{i=15} = 0$). The potential energy function $U_{\text{torsion}}(x)$ represents the full set of backbone amide ω-angle proper and improper torsions, progressively decreased across the replicas, with $\lambda'_{i=0} = 1$ and $\lambda'_{i=15} = 0.60$ (described in more detail below). The scaling of torsion barriers for replicas with weak distance restraints is designed to encourage sampling backbone *cis*/*trans* isomerization throughout the simulation. These torsion barriers are not fully scaled to zero to avoid getting trapped in deep non-physical energy basins (omega angles ≈ 90°, unpublished findings). The function $U^{(0)}(x)$ represents all the energy terms in the force field that do not change across replicas.

We perform HREX simulations as implemented in GROMACS 2021.2, attempting swaps between neighboring replicas every 5 ps. HREX simulations used 16 replicas. For *N*spe$_{7\text{-}1}$ and *N*spe$_{7\text{-}2}$, we perform 1 μs, producing an aggregate 16 μs of trajectory data, and for *N*spe$_{10}$, we stop at 400 ns, which produces an aggregate 6.4 μs of trajectory data in total.

Flat-bottom restraints. The flat-bottom distance restraints used in the HREX have the functional form:

$$V_{dr}(r_{ij}) = \begin{cases} \frac{1}{2} k_{dr}(r_{ij} - r_0)^2 & \text{for } r_{ij} < r_0 \\ 0 & \text{for } r_0 \le r_{ij} < r_1 \\ \frac{1}{2} k_{dr}(r_{ij} - r_1)^2 & \text{for } r_1 \le r_{ij} < r_2 \\ \frac{1}{2} k_{dr}(r_2 - r_1)\left(2r_{ij} - r_2 - r_1\right) & \text{for } r_2 \le r_{ij} \end{cases}$$

Here, r_{ij} represents the distance between two hydrogen atoms i and j, while k_{dr} controls the strength of the restraint (which gets scaled by λ_i). The value of k_{dr} is set to 5000 kJ mol^{-1} nm^{-1}. Parameters r_0, r_1, and r_2 define the shape of the restraint energy function, which are different for strong, medium, and weak restraints (**Table S9**). The restraint potential energy function is visualized for a "strong" restraint in **Figure S14a**.

Scaled omega-angle torsion potentials. Proper omega dihedral potentials for the backbone omega-angles were scaled by λ'_i to encourage *cis*/*trans* isomerization. This was achieved by modifying the amplitude k_φ of the cosine function expansion defining the dihedral potential for the omega angles. The unmodified STEPs force field sets this parameter to k_φ = 10.82505 kJ mol^{-1} (as does GAFF2); in the last replica this value is scaled by 60% to k_φ = 6.49503 kJ mol^{-1}. A visualization of these potentials for various λ'_i are shown in **Figure S14b**.

Optimization of thermodynamic intermediates. To determine the optimal values of λ_i and λ'_i, we link them to scale proportionally via the relation $\lambda'_i = 0.60 + 0.40\ \lambda_i$, and perform preliminary HREX simulations of 1 ns. From these simulations, we collect distributions of $\Delta U_{ij}(x_i) = U_j(x_i) - U_i(x_i)$ values and use the *pylambdaopt* algorithm to determine the spacing of λ_i (and linked λ'_i) values.[27] This algorithm minimizes the thermodynamic length between intermediates, maximizing the overall acceptance rate. Two rounds of initial simulation, followed by optimization by *pylambdaopt*, were required for convergence of the lambda values.

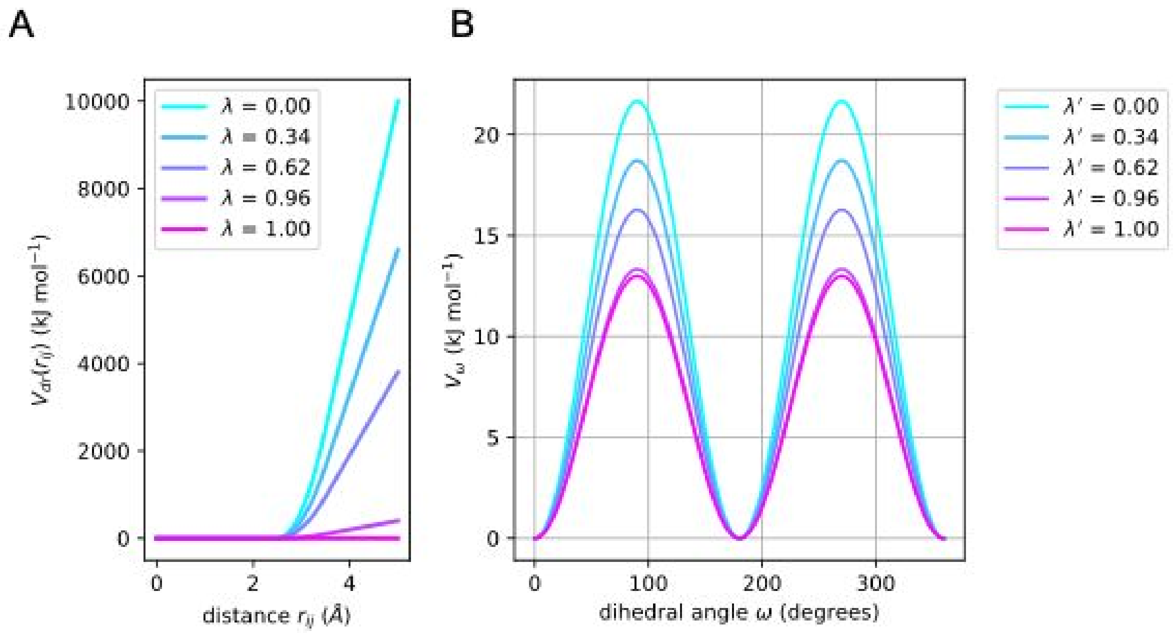

Figure S15. Visualization of restraint potentials. (A) Visualization of a "strong" interproton distance restraint function for several λ_i values. (B) Visualization of the omega-angle proper torsion potential scaled using several λ'_i values.

Table S9. Parameters used to define flat-bottom distance restraints for strong, medium, and weak distance restraints.

	r_0 (Å)	r_i (Å)	r_2 (Å)
strong	0	2.5	3.5
medium	0	3.5	4.5
weak	0	0.5	0.6

Table S10. Summary of free-energy perturbation λ value for each peptoid used in HREX and REMD simulations.

	λ_i values		
Replica index, *i*	***N*spe$_{7\text{-}1}$**	***N*spe$_{7\text{-}2}$**	***N*spe$_{10}$**
0	0	0	0
1	0.11657	0.10864	0.17646
2	0.23463	0.20231	0.32463
3	0.34107	0.29632	0.43983
4	0.43883	0.37865	0.52497
5	0.535	0.46546	0.59829
6	0.61612	0.54693	0.66167
7	0.6812	0.62439	0.71772
8	0.73914	0.69674	0.77195
9	0.79302	0.76203	0.82255
10	0.83985	0.82286	0.86691
11	0.88301	0.87992	0.90586
12	0.92415	0.9228	0.93779
13	0.96051	0.95445	0.96234
14	0.9839	0.97936	0.98252
15	1	1	1

Results. The HREX simulations sample conformations that collectively obey the distance restraints, and find conformations that agree with the putative backbone *cis*/*trans* states assigned based on the cross-peaks seen in the NMR spectroscopy (*N*spe$_{7\text{-}1}$ = *tccttc*, *N*spe$_{7\text{-}2}$ = *tccctc*, *N*spe$_{10}$ = *cctttctc*). Despite this, we found several limitations in the HREX sampling. Even though we optimized the schedule of lambda values, we observed poor exchange that limited the replicas from exploring the full range of thermodynamic states. As a result, the simulations did not fully explore alternative backbone phi-angles. For the *N*spe$_{10}$ specifically, restraints in the HREX simulation produced very distorted omega angles (≈ 90˚) near residues 5 and 6, suggesting poor sampling, leading to frustration in satisfying multiple distance restraints. Examples of competing restraints include long-range restraints between residues 1 and 5, and sets of restraints that can't be mutually satisfied like 6β-5α11 and 6β-6α11, which each favor different side chain chi angles. These observations support the idea that restraints are trapping the molecules in specific conformations that are not the global free energy minimum, motivating our pursuit of a second stage of REMD simulations. Additionally, we found the HREX was not very computationally efficient (50 ns/day), likely due to our implementation of flat-bottom restraints as bonded interactions. The final lambda values are listed in **Table S10**.

(5) Temperature Replica Exchange MD (REMD) simulations

Temperature replica exchange molecular dynamics (REMD) with scaled omega-angle torsion barriers was performed using GROMACS 2021.2[19] with an OpenMPI implementation for parallel simulations. In these simulations, all distance restraints are removed, with simulation replicas having different inverse temperatures $\beta_i = 1/k_B T_i$, and different scaled omega-angle torsion potentials, with reduced potential energy functions defined by $u_i(x) = \beta_i U_i(x) = \beta_i [\ U^{(0)}(x) + \lambda_i' U_{\text{torsion}}(x)\ ]$, and exchange acceptance determined by the Metropolis criterion, $P_{\text{accept}} = \min(1, \exp(\ [\beta_i U_i(x_i) - \beta_i U_i(x_j) + \beta_j U_j(x_j) - \beta_j U_j(x_i)]))$.

The values of λ_i' are kept the same as the HREX simulations, and the temperatures T_i range from 300 K to 450 K. Sixteen replicas were used in the REMD simulations, with spacing determined by the *pylambdaopt* algorithm. We directly use the lambda spacing from HREX. The starting conformations of all peptoids are taken from the HREX simulations after 200 ns. At this point, all the peptoids have the desired backbone *cis*/*trans* amide isomers and remain in these conformations.

REMD was performed with attempted exchanges every 1 ps, achieving efficiencies of 260 ns/day for the *N*spe$_{7\text{-}1}$ and *N*spe$_{7\text{-}2}$, and 230 ns/day for *N*spe$_{10}$. Aggregate trajectory data of 2 μs × 16 replicas = 32 μs were collected for *N*spe$_{7\text{-}1}$ and *N*spe$_{7\text{-}2}$, and 3 μs × 16 replicas = 48 μs for *N*spe$_{10}$.

Results: <u>*Small system sizes enable efficient exchange in explicit-solvent.*</u> One of the known limitations of REMD simulations is poor exchange in explicit-solvent systems. This is because as the number of atoms in a simulation grows, the distribution of $\Delta u_{ij}(x_i) = \beta_j U_j(x) - \beta_i U_i(x)$ values grows away from zero, leading to vanishingly small acceptance probabilities without resorting to large numbers of replicas. Fortunately, because of the small size of our simulation systems, we observe efficient exchange in all systems using only 16 replicas, with average acceptance probabilities of 0.2226, 0.2234, and 0.149 for *N*spe$_{7\text{-}1}$, *N*spe$_{7\text{-}2}$, and *N*spe$_{10}$, respectively. Traces of the thermodynamic index *i* over time for all replicas show efficient exploration of all ensembles (**Figures S15, S16 and S17**).

<u>*REMD simulations show efficient sampling of phi- and chi-angles.*</u> Traces of backbone omega-, phi-, and psi-angles, along with traces of sidechain chi-angles, are shown for *N*spe$_{7\text{-}1}$ (**Figure S18**), *N*spe$_{7\text{-}2}$ (**Figure S19**), and *N*spe$_{10}$ (**Figure S20**). Importantly, for all systems, these traces reveal excellent sampling of backbone phi-angles at both -90˚ and +90˚ the two main minima expected for peptoids.[28] The traces also reveal excellent sampling of sidechain chi-angles at all three expected basins for a sp^3-hybridized bond (trans, gauche+, and gauche). Backbone omega-angles are also heterogeneously sampled. For all systems, backbone psi-angles stay mainly in the expected trans state centered at ψ = 180˚.

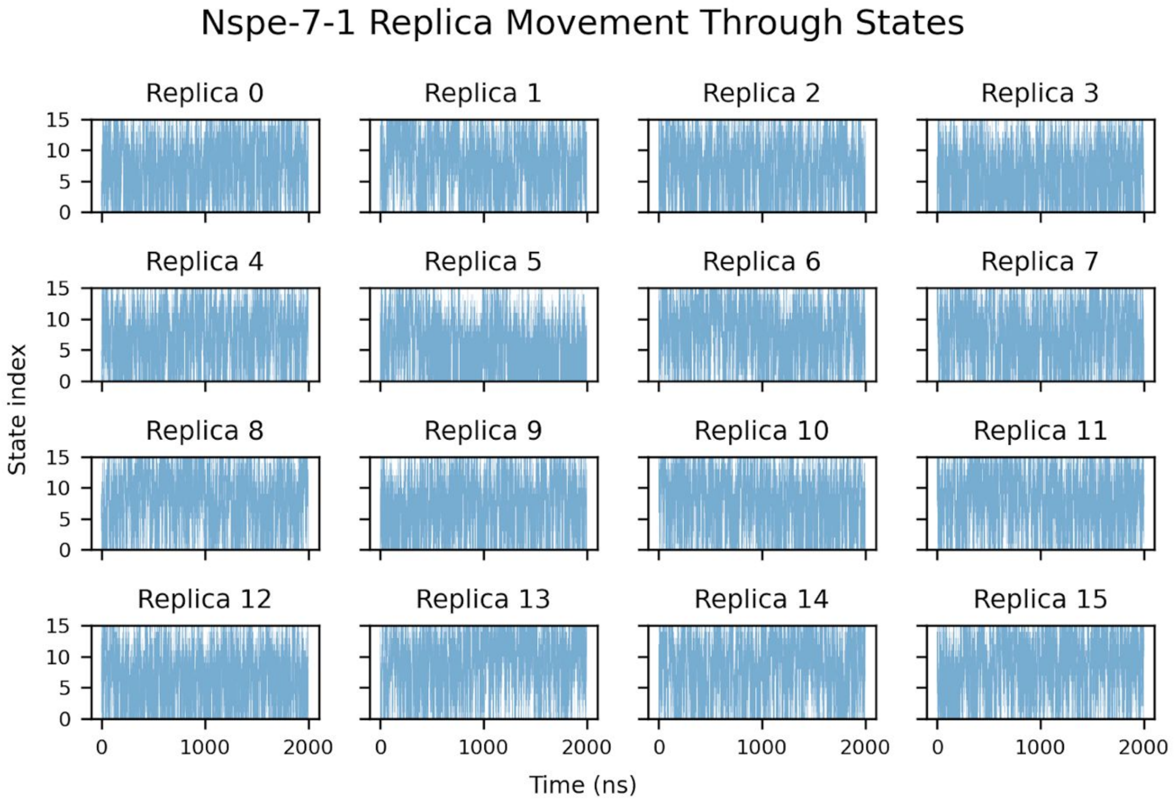

Figure S16. Traces of thermodynamic index *i* over time for REMD simulations of *N*spe$_{7\text{-}1}$.

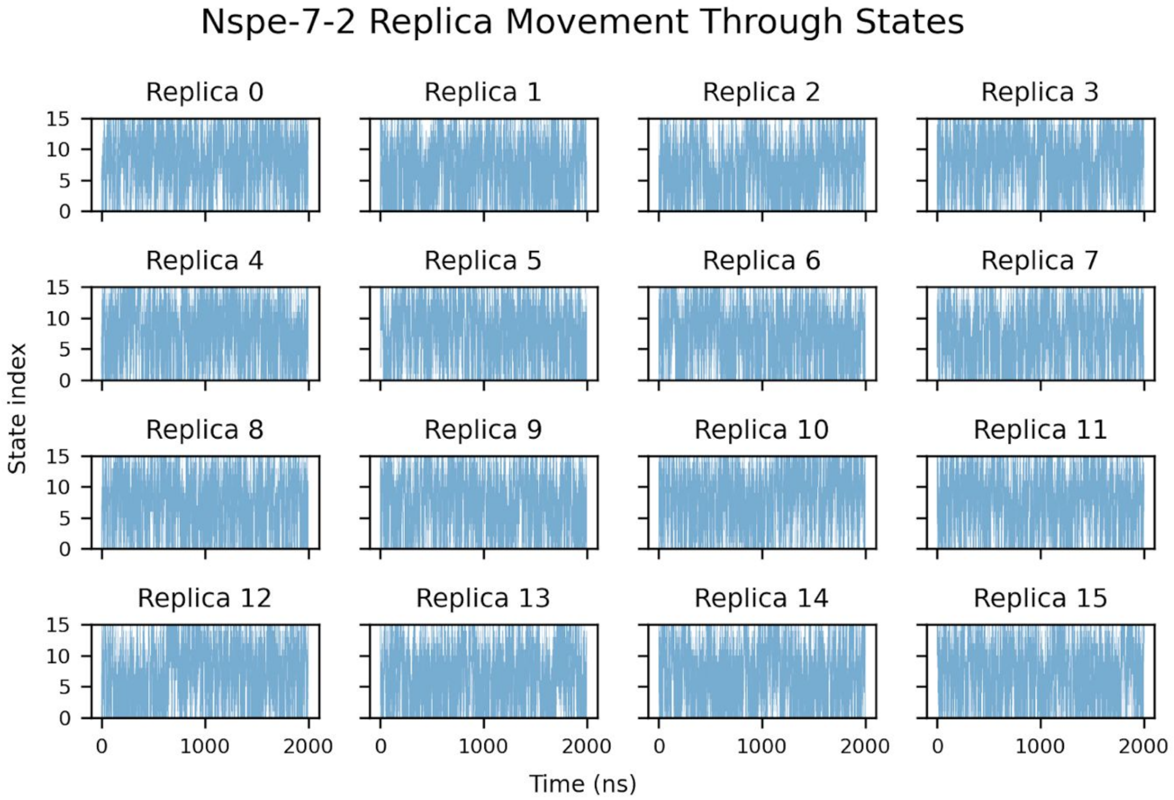

Figure S17. Traces of thermodynamic index *i* over time for REMD simulations of *N*spe$_{7\text{-}2}$.

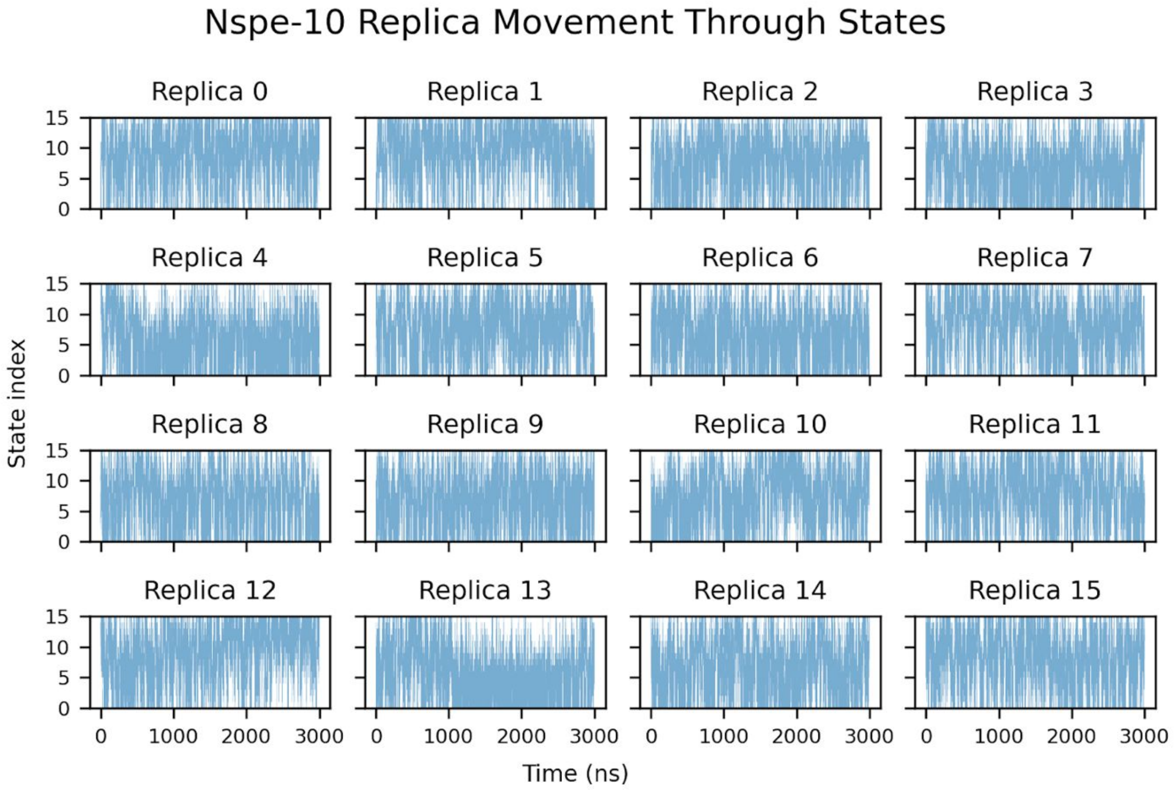

Figure S18. Traces of thermodynamic index i over time for REMD simulations of *N*spe$_{10}$.

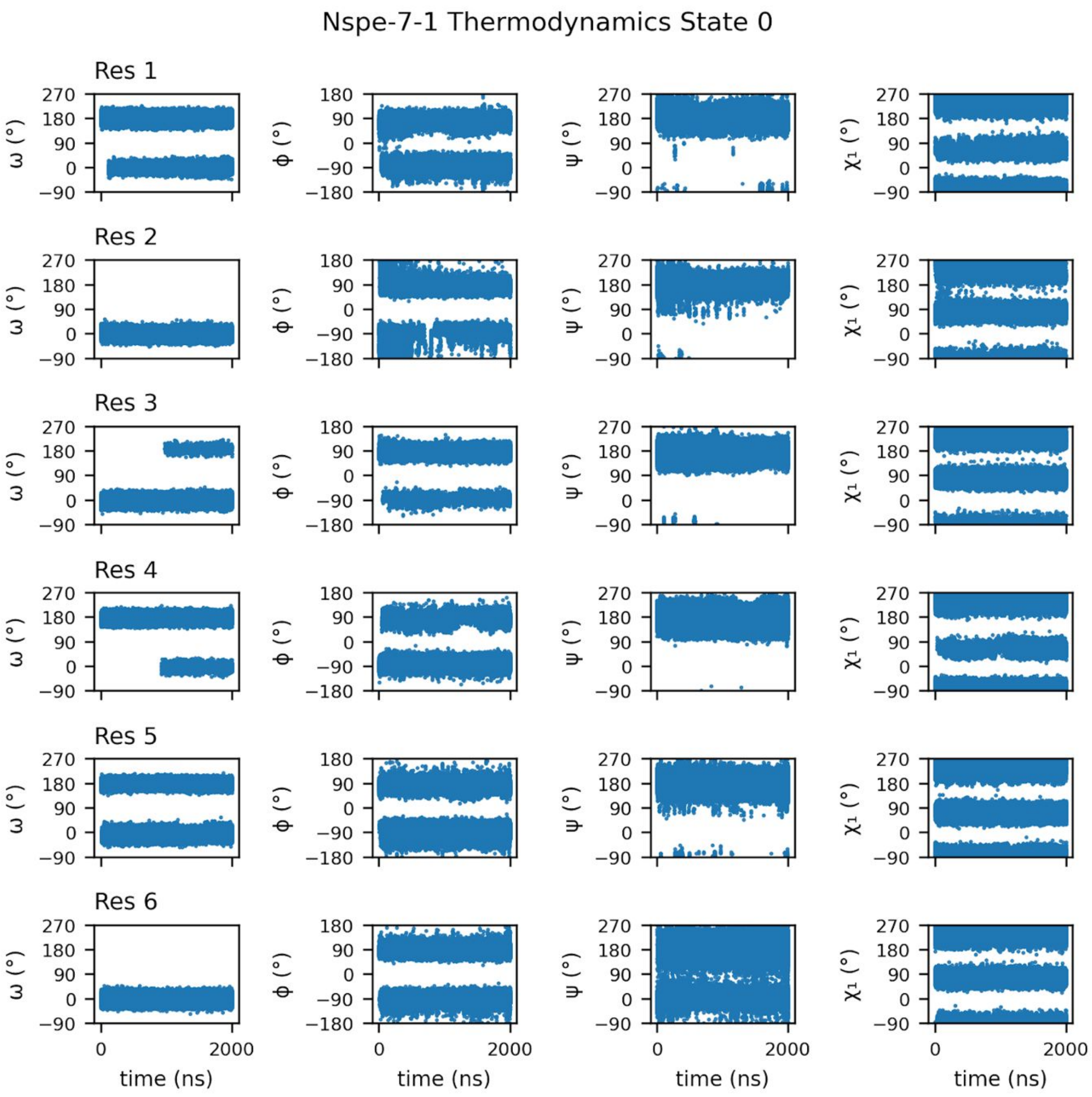

Figure S19. Traces of backbone omega-, phi- and psi-angles, and sidechain chi-angles, observed over time in the REMD simulation for each residue of *N*spe$_{7\text{-}1}$ in the unbiased ensemble at 300 K (replica index *i*=0).

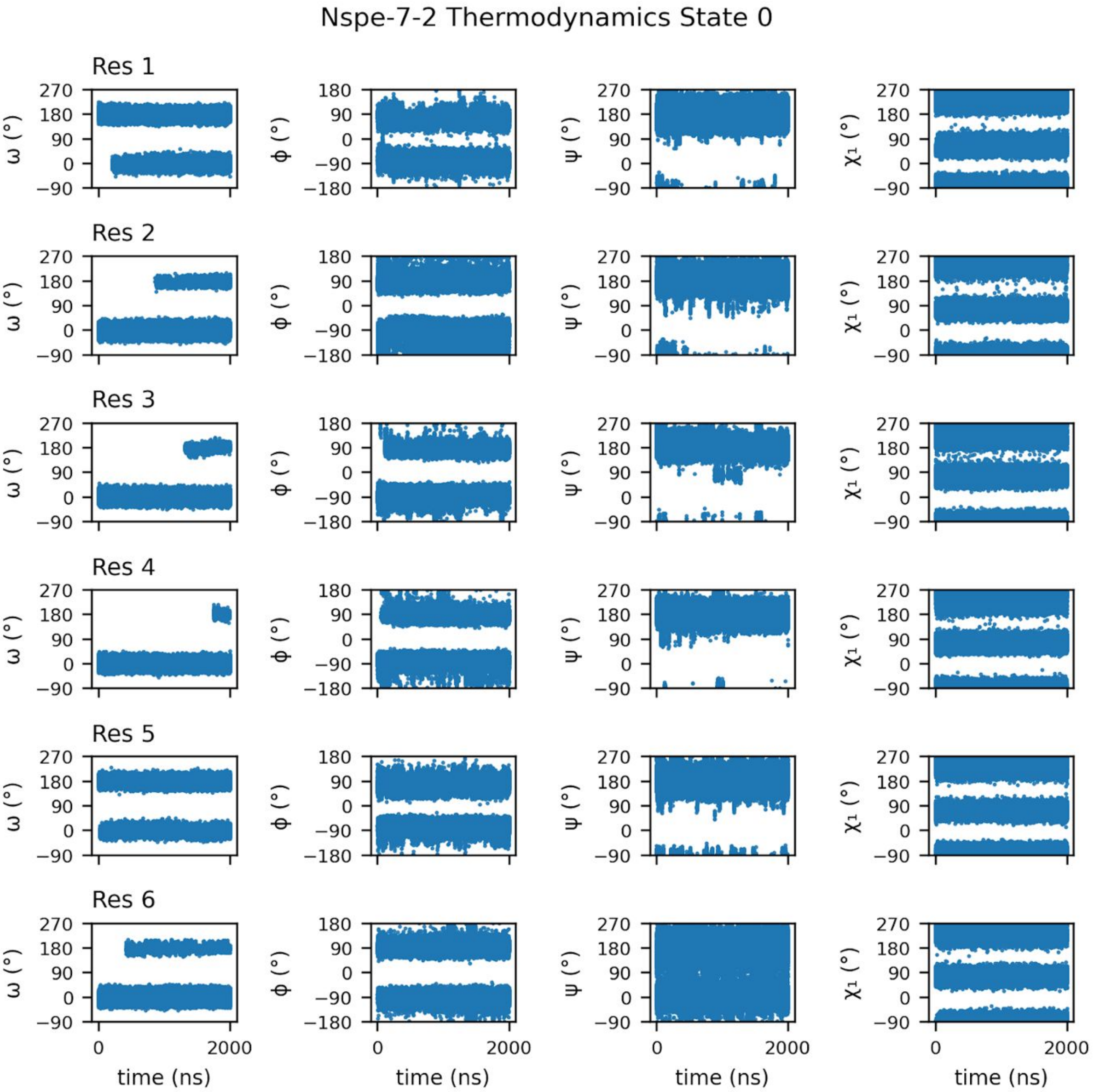

Figure S20. Traces of backbone omega-, phi- and psi-angles, and sidechain chi-angles, observed over time in the REMD simulation, for each residue of *N*spe$_{7\text{-}2}$ in the unbiased ensemble at 300 K (replica index $i = 0$).

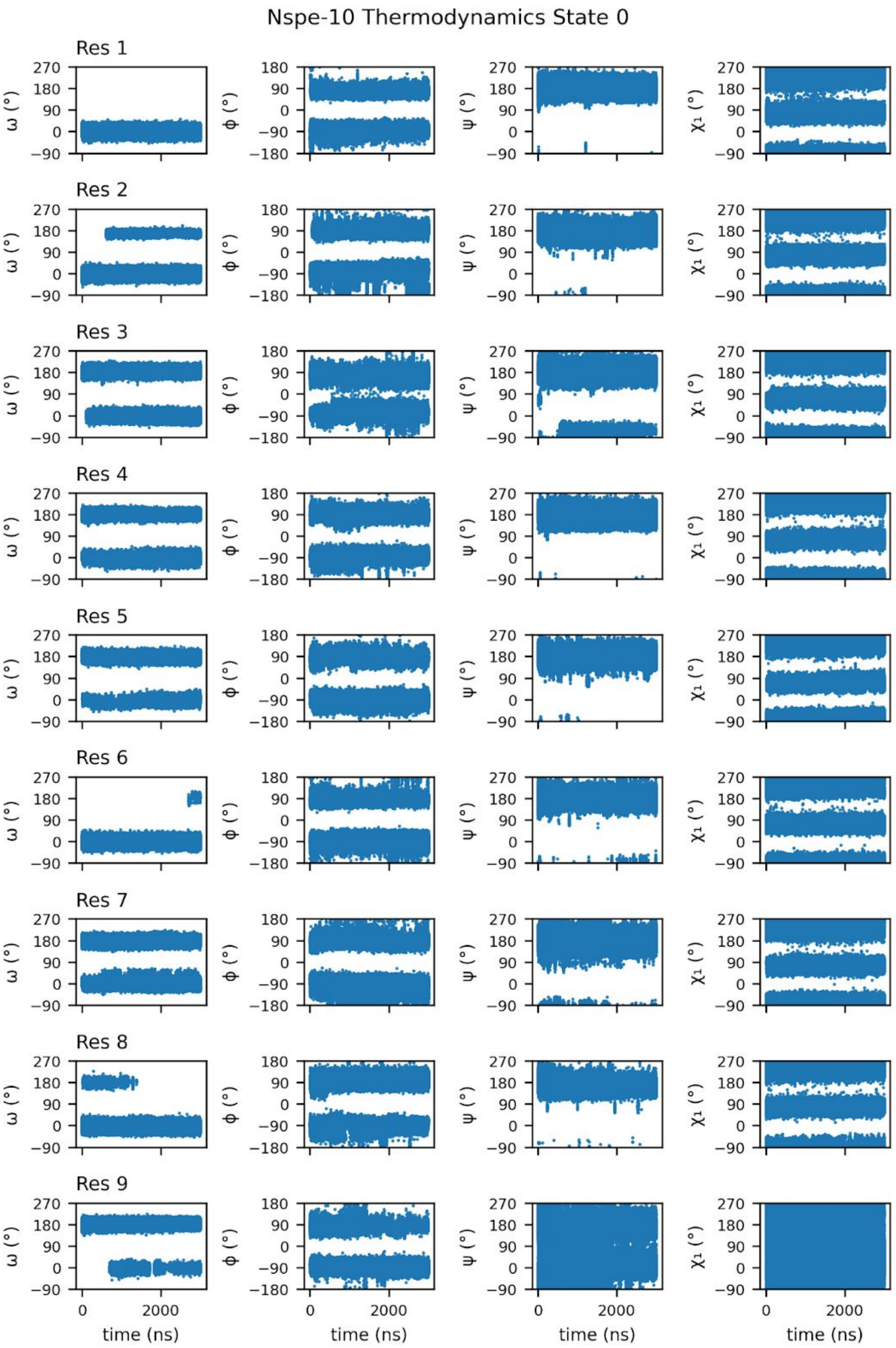

Figure S21. Traces of backbone omega-, phi- and psi-angles, and sidechain chi-angles, observed over time in the REMD simulation, for each residue of *N*spe$_{10}$ in the unbiased ensemble at 300 K (replica index i = 0).

(6) Conformational clustering and BICePs reweighting of simulated ensembles against experimental distance restraints

The REMD replica with index $i = 0$ represents a thermodynamic ensemble at 300 K with unscaled omega-angle torsions; samples from the replica provide a model structural ensemble for the polypeptoids that we use as a starting point for reweighting.

We first examine the heterogeneity of the ensemble by comparing sampled conformations to the distance restraints. To do this, we bin all the conformations sampled by the 300K REMD replica into 92 conformational states for $N\text{spe}_{7\text{-}1}$, 181 conformational states for $N\text{spe}_{7\text{-}2}$, and 356 conformational states for $N\text{spe}_{10}$. All the binned conformational states are defined by unique sets of omega-angles (*cis* vs. *trans*) and phi-angles (+ vs. -). For each bin, we randomly sample up to 50 conformations (if less than 50, then we used all samples in the bins) and from this selection calculate means and variance of all restrained distances (including all pairs of equivalent protons) and compare these values to the experimental distance restraints (**Figure S21**). This comparison is useful because it shows that some distance restraints are highly informative, such as $3^{\alpha 11}$–8^{NH1} for $N\text{spe}_{7\text{-}1}$ and $N\text{spe}_{7\text{-}2}$, and $6^{\alpha 11}$–1^{NH+}, $8^{\alpha 11}$–1^{NH+} and $10^{\alpha 11}$–$1^{\alpha 11}$ for $N\text{spe}_{10}$. All the restraints are long distances, which fall into expectation since they served as indications for folding. The wide distribution of these distances observed across the sampled backbone conformations, suggests that only a small subset are able to satisfy these distance restraints. Other distance restraints are not as informative, in the sense that interproton distances are small regardless of the distance restraints.

Next, we reweighted the populations of these same conformational states (unique sets of omega- and phi-angles) against the experimental distance restraints using the Bayesian Inference of Conformational Populations (BICePs) algorithm.[29–31] The BICePs algorithm uses the simulated populations as a Bayesian prior, along with a likelihood function that enforces experimental restraints, to sample the posterior distribution $P(X,\sigma)$ of conformational populations X and uncertainties σ given ensemble-averaged experimental restraint data D, according to

$$P(X,\sigma) \propto P(D|\, X,\sigma)\, P(X)\, P(\sigma),$$

where $P(D|\, X,\sigma)$ is the likelihood function, $P(X)$ is the prior populations from the REMD simulation, $P(D|\, X,\sigma)$ is the likelihood function, and $P(\sigma) \sim \sigma^{-1}$ is a non-informative Jeffreys prior.

For all systems, we performed BICePs calculations using 1M steps of MCMC sampling. For $N\text{spe}_{7\text{-}1}$ and $N\text{spe}_{7\text{-}2}$, we used a Gaussian likelihood function, proportional to $\exp(-\chi^2/2)$, where χ^2 is a (weighted) sum of squared deviations from the experimental distances, divided by σ^2. For N equivalent pairs of protons, the weight of each squared deviation term is $1/N$. For $N\text{spe}_{10}$, we used the Good-Bad likelihood function, described in Raddi et al. (2025)[30], which is more sensitive to experimental outliers.

For Nspe$_{10}$, we used replica-averaging MCMC to do maximum-entropy reweighting[30] for Nspe$_{10}$, while for Nspe$_{7\text{-}1}$ and Nspe$_{7\text{-}2}$, we only use a single replica, which can be considered a "maximum-parsimony" approach.[32] The reason for this is because we are modeling Nspe$_{7\text{-}1}$ and Nspe$_{7\text{-}2}$ using separate BICePs calculations with separate sets of experimental restraints, which is an inappropriate scenario for the maximum-entropy approach. For Nspe$_{10}$, we use 128 replicas. The type of model and number of replicas used for each system are shown in **Table S11**.

Table S11. BICePs models and parameters used for each system.

	Number of replicas	Likelihood model	MCMC steps
Nspe$_{7\text{-}1}$	1	Gaussian (single-replica)	1,000,000
Nspe$_{7\text{-}2}$	1	Gaussian (single-replica)	1,000,000
Nspe$_{10}$	128	Good-Bad	1,000,000

Results

Summaries of the BICePs results are shown for Nspe$_{7\text{-}1}$ (**Figure S22**), Nspe$_{7\text{-}2}$ (**Figure S23**) and Nspe$_{10}$ (**Figure S24**). In each case, the left panel shows a scatter plot of conformational states, where the horizontal axis is "p_i (exp)" and the vertical axis is "p_i (sim+exp)". The horizontal axis displays the inferred posterior populations in case where the prior populations are uniform (i.e. $P(X) \sim 1$, the case where there is no information from simulations). The vertical axis displays the inferred populations given both simulation and experimental information. Thus, conformational states with large populations that appear above the diagonal are both consistent with the experimental restraints, and predicted to be highly populated in unbiased simulations. On the right panel of these plots are shown posterior distributions of the uncertainty parameter σ. In all cases, the distribution is centered around 1.5 Å, which is reasonable considering the assumptions made in deriving distance restraints from the NOE cross-peaks.

Finally, to derive a small set (~ 10) of molecular conformations that exemplify the most populated state, we select the highest-population state determined by BICePs, and from that state select ten individual conformations with the smallest weighted root mean-squared deviations (wRMSDs) from the experimental distance restraints. The weighted RMSD is calculated as

$$\text{wRMSD} = \sqrt{\frac{\sum_{k=1}^{K} w_k\left(d_k - d_k^{\text{ref}}\right)^2}{\sum_{k=1}^{K} w_k}},$$

where the index k=1,...,K specifies each of the K distance restraints, d_k is the kth model distance, d_k^{ref} is the kth experimental reference distance, and $w_k = 1/N$ is the weight for the kth distance corresponding to

a proton pair that is one of N equivalents. The values of d_k^{ref} reference distances are 2.5 Å for strong restraints, 3.5 Å for medium restraints, and 5 Å for weak restraints.

A summary of the three conformational states with the largest BICePs-reweighted populations is given in **Table S12**. Finally, we visualize and structurally characterize exemplar conformations for the top-ranked conformational state for *N*spe$_{7\text{-}1}$ (**Figure S25**), *N*spe$_{7\text{-}2}$ (**Figure S26**), and *N*spe$_{10}$ (**Figure S27**).

Table S12. The three conformational states with the largest BICePs-reweighted populations, for each system. Backbone conformations are given as a pattern of *cis* (c) and *trans* (t) omega angles, and corresponding phi-angles (+)

	*N*spe$_{7\text{-}1}$		*N*spe$_{7\text{-}2}$		*N*spe$_{10}$	
Rank	BICePs pop (%)	Backbone conformation	BICePs pop (%)	Backbone conformation	BICePs pop (%)	Backbone conformation
1	**55.4 %**	ω tccttc<br>ϕ -++---	**85.3 %**	**ω tccctc**<br>**ϕ -+----**	**55.9**	**ω cctttctct**<br>**ϕ --+------**
2	21.4 %	ω tccttc<br>ϕ +-+---	4.2 %	ω tccctc<br>ϕ +-++++	22.6	ω cctctccct*<br>ϕ +++++-++-
3	18.5 %	ω tccttc<br>ϕ +-+-++	1.7 %	ω tccctc<br>ϕ +---++	12.1	ω cctttctct<br>ϕ --+-++--+

**cis/trans* pattern inconsistent with the NMR; not a valid solution.

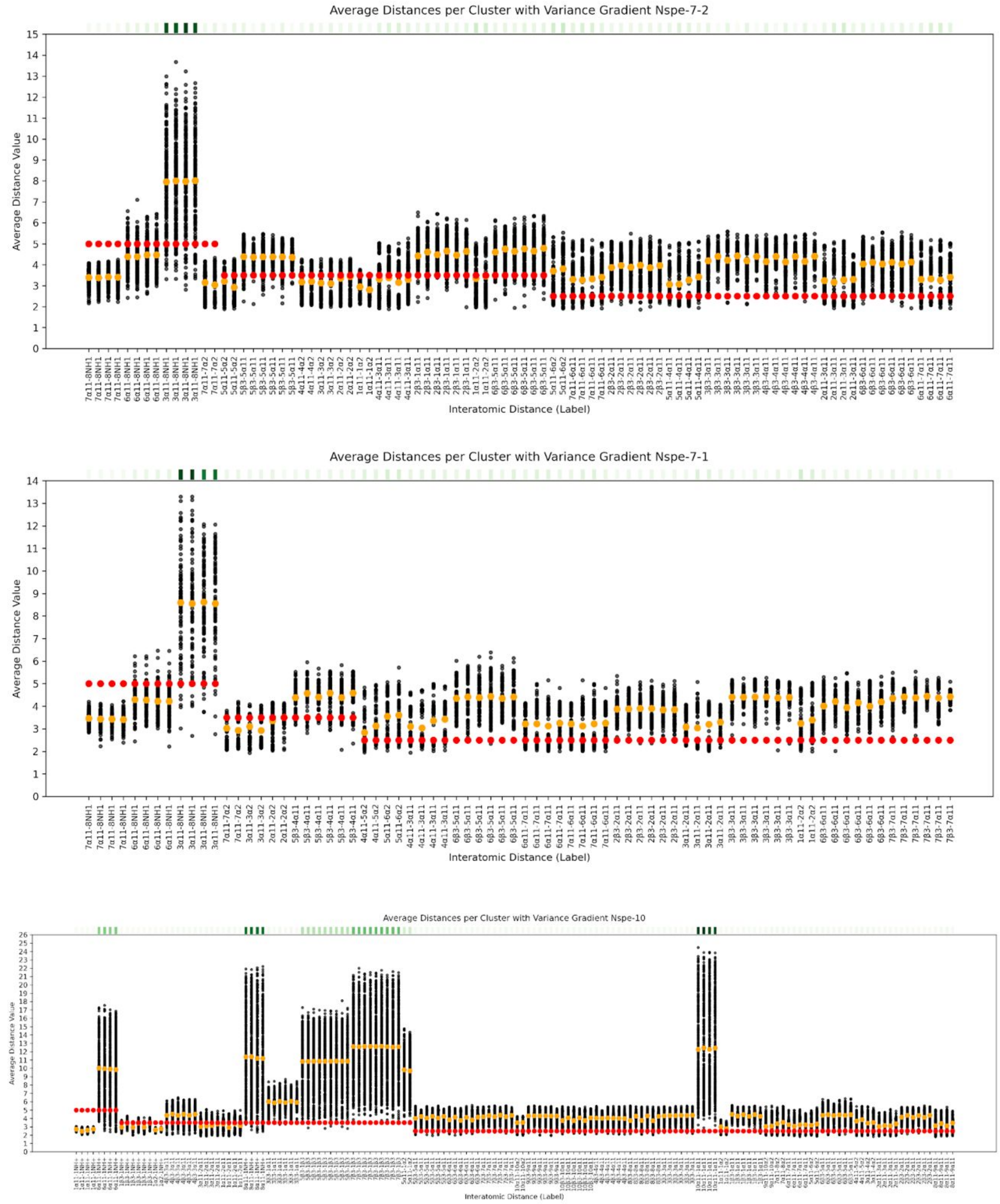

Figure S22. Mean distances (yellow dots) and variances (green) across conformational states binned by unique sets of omega- and phi-angles, compared to the experimental restraint distances (red dots), for *N*spe$_{7\text{-}1}$ (top panel), *N*spe$_{7\text{-}2}$ (middle panel), and *N*spe$_{10}$ (bottom panel).

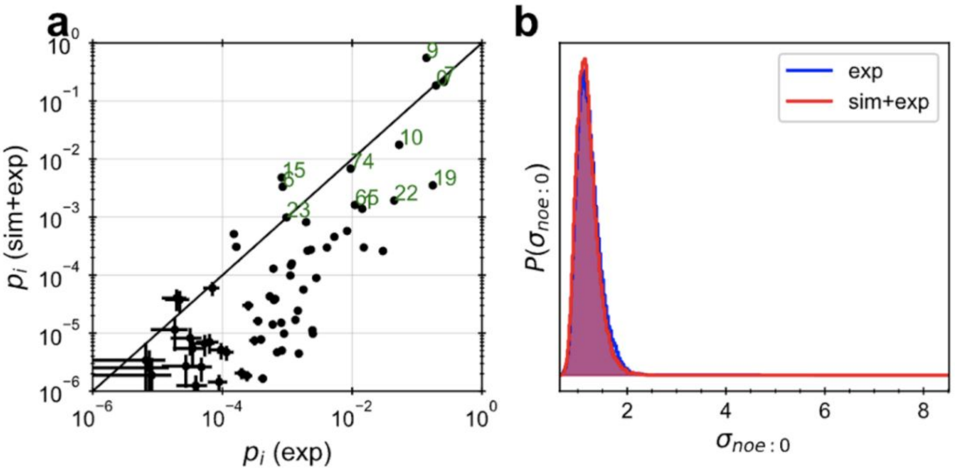

Figure S23. Summaries of the BICePs results for *N*spe$_{7\text{-}1}$. (a) Scatter plots of predicted conformational state populations "p_i (exp)" using uniform prior populations, versus "p_i (sim+exp)" using simulated populations from the REMD simulations. Conformational states with large populations that appear above the diagonal are both consistent with the experimental restraints, and predicted to be highly populated in unbiased simulations. (b) Posterior distributions of the uncertainty parameter σ.

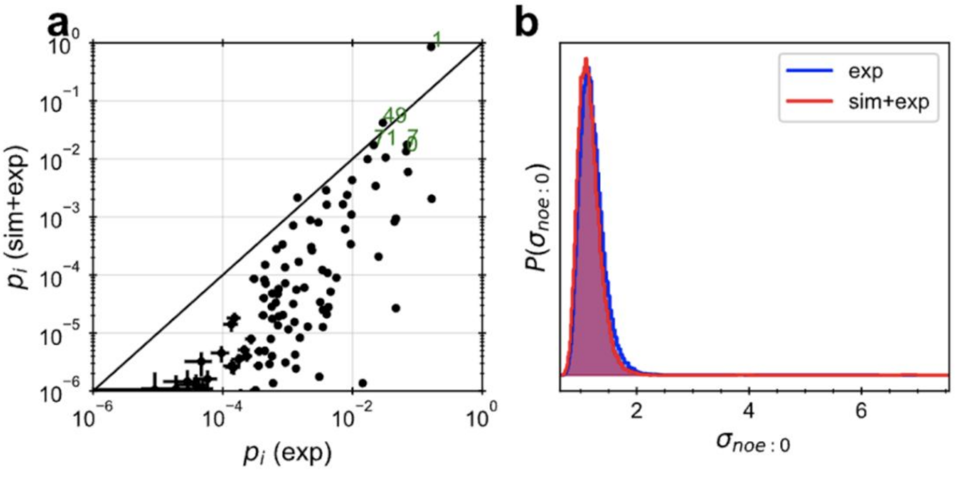

Figure S24. Summaries of the BICePs results for *N*spe$_{7\text{-}2}$. (a) Scatter plots of predicted conformational state populations "p_i (exp)" using uniform prior populations, versus "p_i (sim+exp)" using simulated populations from the REMD simulations. Conformational states with large populations that appear above the diagonal are both consistent with the experimental restraints, and predicted to be highly populated in unbiased simulations. (b) Posterior distributions of the uncertainty parameter σ.

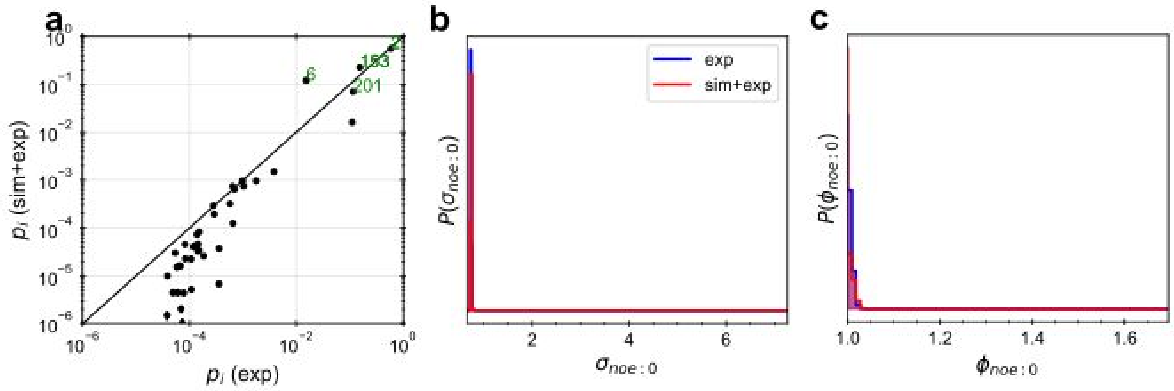

Figure S25. Summaries of the BICePs results for Nspe10. (a) Scatter plots of predicted conformational state populations "p_i (exp)" using uniform prior populations, versus "p_i (sim+exp)" using simulated populations from the REMD simulations. Conformational states with large populations that appear above the diagonal are both consistent with the experimental restraints, and predicted to be highly populated in unbiased simulations. (b) Posterior distributions of the uncertainty parameter σ. (c) Posterior distributions of the uncertainty parameter φ.

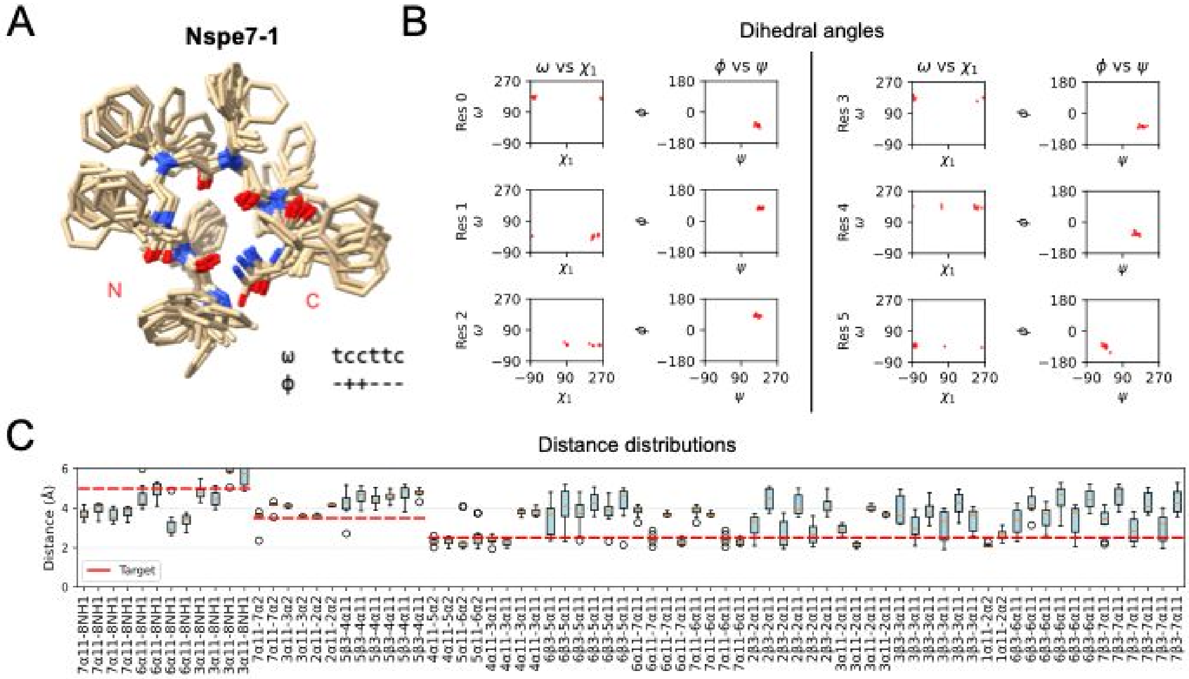

Figure S26. Structural characterization of the most populated conformational state for *N*spe$_{7-1}$ predicted by BICePs. (A) Molecular visualization of ten exemplar conformations, annotated with the pattern of backbone omega- and phi-angles, and *N*- and *C*-termini (red). (B) Scatter plots of dihedral angles (ω vs. χ_1, and φ vs. ψ) of the ten exemplar conformations, for each residue. (C) Box-and-whisker plots illustrating the distribution of each interproton distance for this conformational state. Each box shows the interquartile range (IQR, from quartile 1 to 3) corresponding to 50% of the data about the mean (orange line). The whiskers extend from the box to the farthest data points lying within 1.5 times the IQR from the box.

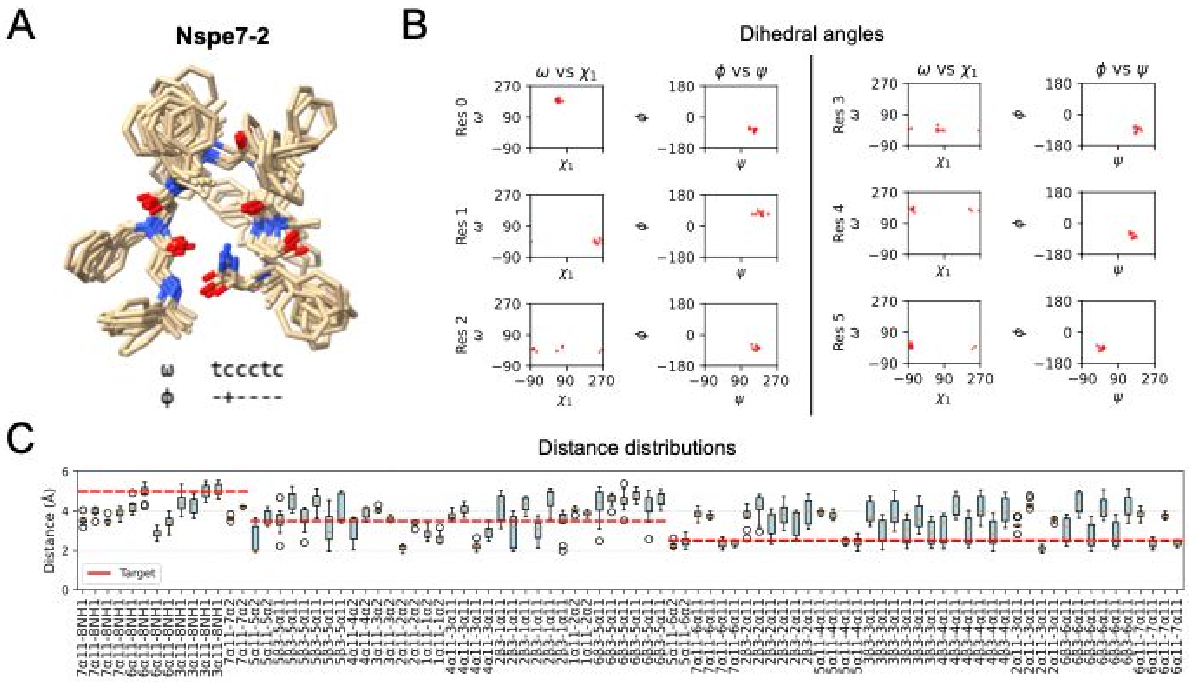

Figure S27. Structural characterization of the most populated conformational state for *N*spe$_{7\text{-}2}$ predicted by BICePs. (A) Molecular visualization of ten exemplar conformations, annotated with the pattern of backbone omega- and phi-angles. (B) Scatter plots of dihedral angles (ω vs. χ_1, and φ vs. ψ) of the ten exemplar conformations, for each residue. (C) Box-and-whisker plots illustrating the distribution of each interproton distance for this conformational state. Each box shows the interquartile range (IQR, from quartile 1 to 3) corresponding to 50% of the data about the mean (orange line). The whiskers extend from the box to the farthest data points lying within 1.5 times the IQR from the box.

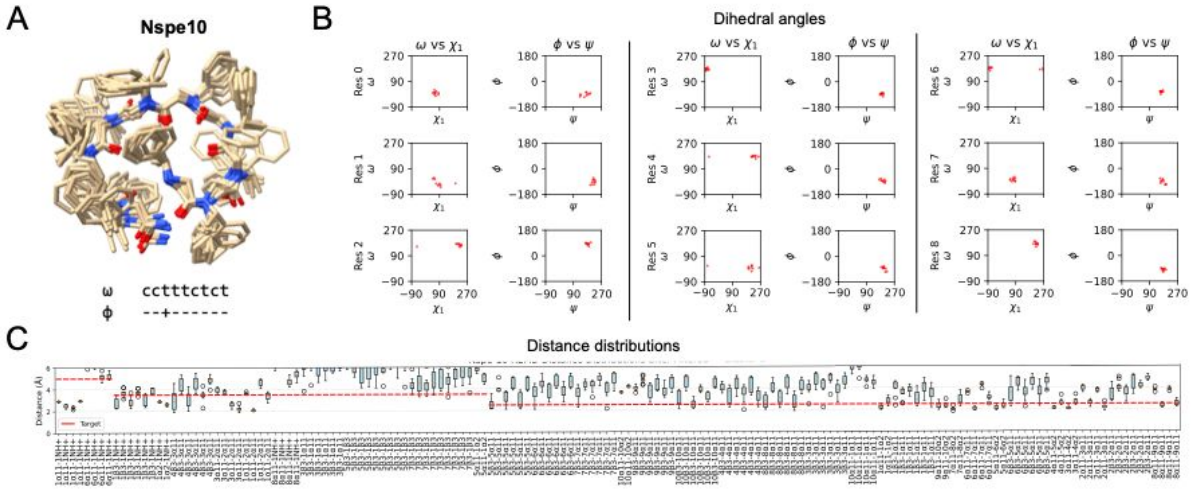

Figure S28. Structural characterization of the most populated conformational state for *N*spe$_{10}$ predicted by BICePs. (A) Molecular visualization of ten exemplar conformations, annotated with the pattern of backbone omega- and phi-angles. (B) Scatter plots of dihedral angles (ω vs. χ_1, and φ vs. ψ) of the ten exemplar conformations, for each residue. (C) Box-and-whisker plots illustrating the distribution of each interproton distance for this conformational state. Each box shows the interquartile range (IQR, from quartile 1 to 3) corresponding to 50% of the data about the mean (orange line). The whiskers extend from the box to the farthest data points lying within 1.5 times the IQR from the box.

7. References

(1) Zuckermann, R. N.; Kerr, J. M.; Kent, S. B. H.; Moos, W. H. *J. Am. Chem. Soc.* **1992**, *114*, 10646-10647.

(2) Aue, W. P.; Bartholdi, E.; Ernst, R. R. Two-dimensional spectroscopy. Application to nuclear magnetic resonance. *J. Chem. Phys.* **1976**, *64*, 2229-2246.

(3) Schleucher, J.; Schwendinger, M.; Sattler, M.; Schmidt, P.; Schedletzky, O.; Glaser, S. J.; Sørensen, O. W.; Griesinger, C. A general enhancement scheme in heteronuclear multidimensional NMR employing pulsed field gradients. *J. Biomol. NMR* **1994**, *4*, 301-306.

(4) Kay, L. E.; Keifer, P.; Saarinen, T. Pure absorption gradient enhanced heteronuclear single quantum correlation spectroscopy with improved sensitivity. *J. Am. Chem. Soc.* **1992**, *114*, 10663-10665.

(5) Palmer, A. G., III; Cavanagh, J.; Wright, P. E.; Rance, M. Sensitivity improvement in proton-detected two-dimensional heteronuclear correlation NMR spectroscopy. *J. Magn. Reson.* **1991**, *93*, 151-170.

(6) Bax, A.; Summers, M. F. ^{1}H and ^{13}C assignments from sensitivity-enhanced detection of heteronuclear multiple-bond connectivity by 2D multiple-quantum NMR. *J. Am. Chem. Soc.* **1986**, *108*, 2093–2094.

(7) Willker, W.; Leibfritz, D.; Kerssebaum, R.; Bermel, W. Gradient selection in inverse heteronuclear correlation spectroscopy. *Magn. Reson. Chem.* **1993**, *31*, 287–292.

(8) Bax, A.; Davis, D. G. Practical aspects of two-dimensional transverse NOE spectroscopy. *J. Magn. Reson.* **1985**, *63*, 207–213.

(9) Hwang, T.-L.; Shaka, A. J. Cross relaxation without TOCSY: transverse rotating-frame Overhauser effect spectroscopy. *J. Am. Chem. Soc.* **1992**, *114*, 3157–3159.

(10) Lee, W.; Rahimi, M.; Lee, Y.; Chiu, A. POKY: A Software Suite for Multidimensional NMR and 3D Structure Calculation of Biomolecules. *Bioinformatics* **2021**, *37*, 3041-3042.

(11) Lodewyk, M. W.; Siebert, M. R.; Tantillo, D. J. Computational Prediction of 1H and 13C Chemical Shifts: A Useful Tool for Natural Product, Mechanistic, and Synthetic Organic Chemistry. *Chem. Rev.* **2012**, *112* (3), 1839–1862.

(12) Flaig, D.; Maurer, M.; Hanni, M.; Braunger, K.; Kick, L.; Thubauville, M.; Ochsenfeld, C. Benchmarking Hydrogen and Carbon NMR Chemical Shifts at HF, DFT, and MP2 Levels. *J. Chem. Theory Comput.* **2014**, *10* (2), 572–578.

(13) Frisch, M. J.; et al. Gaussian 16 Rev. C.01, 2016.

(14) Rezai, T.; Yu, B.; Millhauser, G. L.; Jacobson, M. P.; Lokey, R. S. Testing the conformational hypothesis of passive membrane permeability using synthetic cyclic peptide diastereomers. *J. Am. Chem. Soc.* **2006**, *128*, 2510−2511.

(15) Furukawa, A.; Townsend, C. E.; Schwochert, J.; Pye, C. R.; Bednarek, M. A.; Lokey, R. S. Passive membrane permeability in cyclic peptomer scaffolds is robust to extensive variation in side chain functionality and backbone geometry. *J. Med. Chem.* **2016**, *59*, 9503−9512.

(16) Hewitt, W. M.; Leung, S. S. F.; Pye, C. R.; Ponkey, A. R.; Bednarek, M.; Jacobson, M. P.; Lokey, R. S. Cell-Permeable Cyclic Peptides from Synthetic Libraries Inspired by Natural Products. *J. Am. Chem. Soc.* **2015**, *137*, 715–721.

(17) Sebastiano, M. R.; Doak, B. C.; Backlund, M.; Poongavanam, V.; Over, B.; Ermondi, G.; Caron, G.; Matsson, P.; Kihlberg, J. Impact of Dynamically Exposed Polarity on Permeability and Solubility of Chameleonic Drugs Beyond the Rule of 5. *J. Med. Chem.* **2018**, *61*, 4189–4202.

(18) Whitty, A.; Zhong, M.; Viarengo, L.; Beglov, D.; Hall, D. R.; Vajda, S. Quantifying the Chameleonic Properties of Macrocycles and Other High-Molecular-Weight Drugs. *Drug Discovery Today* **2016**, *21*, 712–717.

(19) Abraham, M. J.; Murtola, T.; Schulz, R.; Páll, S.; Smith, J. C.; Hess, B.; Lindahl, E. GROMACS: High Performance Molecular Simulations through Multi-Level Parallelism from Laptops to Supercomputers. *SoftwareX* **2015**, *1–2*, 19–25.

(20) Harris, B. S.; Bejagam, K. K.; Baer, M. D. Development of a Systematic and Extensible Force Field for Peptoids (STEPs). *J. Phys. Chem. B* **2023**, *127* (29), 6573–6584.

(21) Wang, J.; Wolf, R. M.; Caldwell, J. W.; Kollman, P. A.; Case, D. A. Development and Testing of a General Amber Force Field. *J. Comput. Chem.* **2004**, *25* (9), 1157–1174.

(22) Case, D. A.; Aktulga, H. M.; Belfon, K.; Cerutti, D. S.; Cisneros, G. A.; Cruzeiro, V. W. D.; Forouzesh, N.; Giese, T. J.; Götz, A. W.; Gohlke, H.; Izadi, S.; Kasavajhala, K.; Kaymak, M. C.; King, E.; Kurtzman, T.; Lee, T.-S.; Li, P.; Liu, J.; Luchko, T.; Luo, R.; Manathunga, M.; Machado, M. R.; Nguyen, H. M.; O'Hearn, K. A.; Onufriev, A. V.; Pan, F.; Pantano, S.; Qi, R.; Rahnamoun, A.; Risheh, A.; Schott-Verdugo, S.; Shajan, A.; Swails, J.; Wang, J.; Wei, H.; Wu, X.; Wu, Y.; Zhang, S.; Zhao, S.; Zhu, Q.; Cheatham, T. E.; Roe, D. R.; Roitberg, A.; Simmerling, C.; York, D. M.; Nagan, M. C.; Merz, K. M. AmberTools. *J. Chem. Inf. Model.* **2023**, *63* (20), 6183–6191.

(23) He, X.; Man, V. H.; Yang, W.; Lee, T. S.; Wang, J. A Fast and High-Quality Charge Model for the Next Generation General AMBER Force Field. *J. Chem. Phys.* **2020**, *153* (11).

(24) Jakalian, A.; Jack, D. B.; Bayly, C. I. Fast, Efficient Generation of High-Quality Atomic Charges. AM1-BCC Model: II. Parameterization and Validation. *J. Comput. Chem.* **2002**, *23* (16), 1623–1641.

(25) Goold, S.; Raddi, R. M.; Voelz, V. A. Expanded Ensemble Predictions of Toluene–Water Partition Coefficients in the SAMPL9 LogP Challenge. *Phys. Chem. Chem. Phys.* **2025**, *27*, 6005–6013.

(26) Basconi, J. E.; Shirts, M. R. Effects of Temperature Control Algorithms on Transport Properties and Kinetics in Molecular Dynamics Simulations. *J. Chem. Theory Comput.* **2013**, *9* (7), 2887–2899.

(27) Novack, D.; Raddi, R. M.; Zhang, S.; Hurley, M. F. D.; Voelz, V. A. Simple Method to Optimize the Spacing and Number of Alchemical Intermediates in Expanded Ensemble Free Energy Calculations. *J. Chem. Inf. Model.* **2025**, *65* (12), 6089–6101.

(28) Eastwood, J. R. B.; Weisberg, E. I.; Katz, D.; Zuckermann, R. N.; Kirshenbaum, K. Guidelines for Designing Peptoid Structures: Insights from the Peptoid Data Bank. *Pept. Sci.* **2023**, e24307.

(29) Raddi, R. M.; Ge, Y.; Voelz, V. A. BICePs v2.0: Software for Ensemble Reweighting Using Bayesian Inference of Conformational Populations. *J. Chem. Inf. Model.* **2023**, *63* (8), 2370–2381.

(30) Raddi, R. M.; Marshall, T.; Ge, Y.; Voelz, V. A. Model Selection Using Replica Averaging with Bayesian Inference of Conformational Populations. *J. Chem. Theory Comput.* **2025**, *21* (12), 5880–5889.

(31) Voelz, V. A.; Ge, Y.; Raddi, R. M. Reconciling Simulations and Experiments with BICePs: A Review. *Front. Mol. Biosci.* **2021**, *8*, 661520.

(32) Bonomi, M.; Heller, G. T.; Camilloni, C.; Vendruscolo, M. Principles of Protein Structural Ensemble Determination. *Curr. Opin. Struct. Biol.* **2017**, *42*, 106–116.
